# Inhibition of hepatocellular carcinoma growth by blockade of glycosphingolipid synthesis

**DOI:** 10.18632/oncotarget.22648

**Published:** 2017-11-24

**Authors:** Richard Jennemann, Giuseppina Federico, Daniel Mathow, Mariona Rabionet, Francesca Rampoldi, Zoran V. Popovic, Martina Volz, Thomas Hielscher, Roger Sandhoff, Hermann-Josef Gröne

**Affiliations:** ^1^ Department of Cellular and Molecular Pathology, German Cancer Research Center, Heidelberg, Germany; ^2^ Lipid Pathobiochemistry Group, German Cancer Research Center, Heidelberg, Germany; ^3^ Division of Biostatistics, German Cancer Research Center, Heidelberg, Germany

**Keywords:** hepatocellular carcinoma, cytokinesis, glycolipid, glycosphingolipid, sphingomyelin

## Abstract

Hepatocellular carcinoma (HCC) is one of the most frequent cancers. *In vitro* studies suggest that growth and response to therapy of human carcinomas may depend on glycosphingolipid (GSL) expression. Glucosylceramide synthase (GCS), encoded by the gene *Ugcg*, is the basic enzyme required for the synthesis of GSLs. Gene array analysis implied that *Ugcg* is significantly overexpressed in human HCC as compared to non-tumorous liver tissue. Therefore we have investigated whether tumor - genesis and - growth is altered in the absence of GSLs. An endogenous liver cancer model has been initiated by application of diethylnitrosamine in mice lacking *Ugcg* specifically in hepatocytes. We have now shown that hepatocellular tumor initiation and growth in mice is significantly inhibited by hepatic GSL deficiency *in vivo*. Neither the expression of cell cycle proteins, such as cyclins and pathways such as the MAP-kinase/Erk pathway nor the mTOR/Akt pathway as well as the number of liver infiltrating macrophages and T cells were essentially changed in tumors lacking GSLs. Significantly elevated bi-nucleation of atypical hepatocytes, a feature for impaired cytokinesis, was detected in tumors of mice lacking liver-specific GSLs. A reduction of proliferation and restricted growth of tumor microspheres due to delayed, GSL-dependent cytokinesis, analogous to the histopathologic phenotype *in vivo* could be demonstrated *in vitro*. GSL synthesis inhibition may thus constitute a potential therapeutic target for hepatocellular carcinoma.

## INTRODUCTION

Hepatocellular carcinoma (HCC) is the fifth most common form of cancer [1]. Besides hepatitis C virus infection and non-alcoholic steatohepatitis (NASH) [2] environmental toxins such as nitrosamines *e.g.* diethylnitrosamines (DEN) potentially occurring in preserved meat, beer, and in tobacco smoke are relevant risk factors for HCC.

Glucosylceramide synthase (GCS) has been described to be significantly elevated in numerous human cancers including breast, cervix, colon [3], non-small cell lung cancer [4], and papillary thyroid carcinoma [5]. Its overexpression was correlated with chemoresistance [6-9]. Therefore, GCS has been considered as potential target to overcome chemoresistance in cancer cells [10-14]. In addition, GCS and its synthesis products *i.e.* glycosphingolipids (GSLs) may also contribute to carcinogenesis as suggested by *in vitro* cell culture experiments and xenograft cancer models [15-32]. The pathogenetic role of GCS in HCC *in vivo* however is largely unknown.

GCS catalyzes the first step of glucosylceramide (GlcCer) derived GSL synthesis and is encoded by the gene UDP-glucose ceramide glucosyltransferase (*Ugcg*) [33]. GSLs are constituents of all eukaryotic cell membranes predominantly located on the outer leaflet of the cellular plasma membrane. GSLs affect cell adhesion [34, 35] and are involved in intracellular protein and lipid trafficking [36-38]. GSLs also participate in signaling events [39]; they concentrate in lipid domains [40] and modify receptor activity of insulin- [41-43], leptin- [44], and epidermal growth factor receptor (EGFR) [45-47].

In the present study we have verified that glucosylceramide synthase was overexpressed in human HCC. With that information, we addressed the key issue of our investigations whether targeting of *Ugcg* in hepatocytes accompanied by GSL depletion would affect hepatic carcinogenesis *in vivo*. Cell culture is a common well defined tool to solve molecular cell biologic mechanisms, but, these data cannot automatically be translated to complex pathophysiological processes such as cancer development. Therefore, an endogenous tumor model in mice was used in combination with genetic deletion of *Ugcg* in hepatocytes [48]. We have found a significant delay in growth of diethylnitosamine (DEN)-induced liver carcinomas upon *Ugcg* deletion *in vivo*; this could be ascribed to delayed cytokinesis.

## RESULTS

### Gene array expression analysis indicates a significant increase of *UGCG* in human HCC as compared to non-tumorous liver or tissue of healthy organ donors

GCS has been described to be significantly elevated in numerous human cancers [3-5]. The expression of *UGCG* in human hepatocellular carcinoma has not been elucidated so far. For this end, two publicly available GEO HCC datasets with Affymetrix microarray measurements GSE14520 [49, 50] and GSE64041 [51] were used. Normalized and log2-transformed expression values of tumor, non-tumor and healthy donor samples were analyzed.

**Figure 1:**
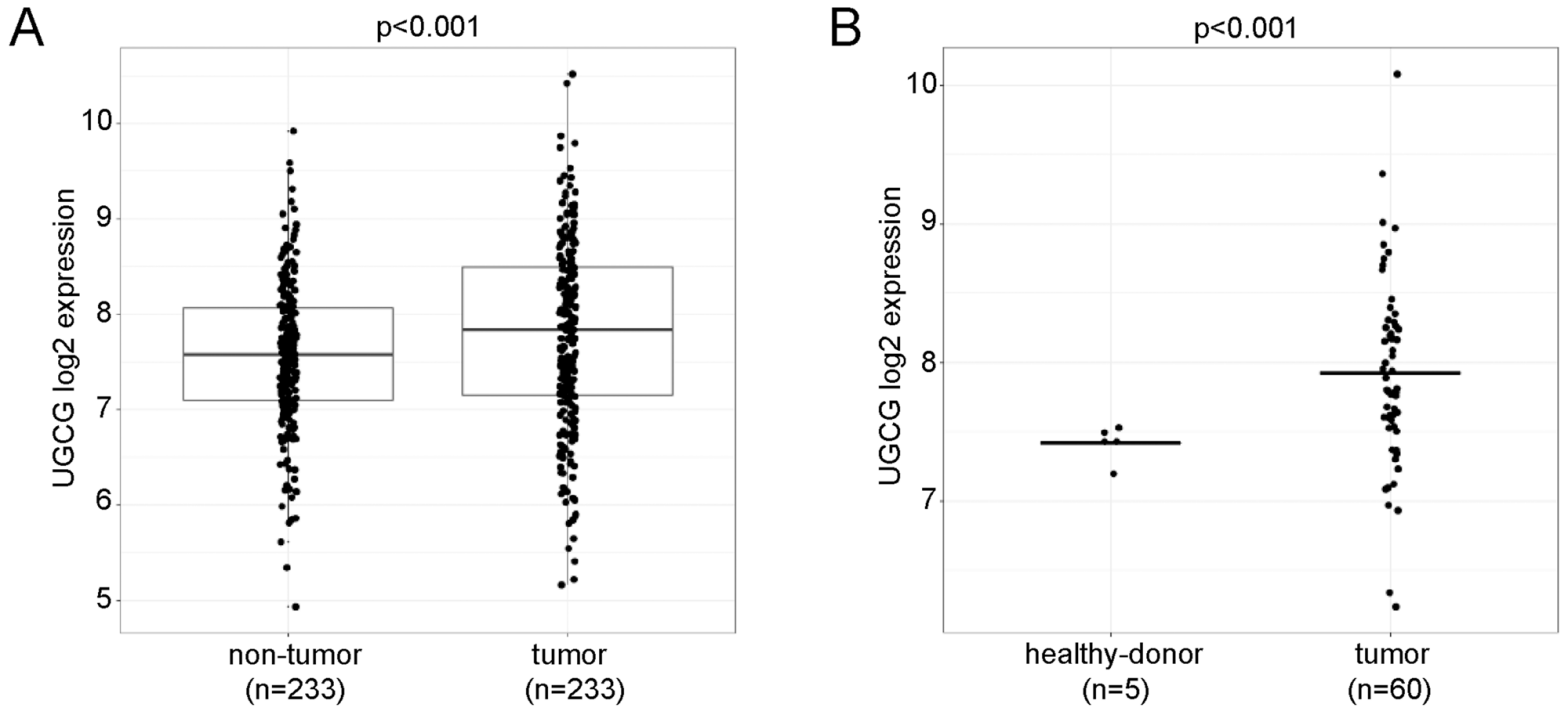
*UGCG* expression analysis in human HCC, non-tumor, and healthy donors **(A)**, *UGCG* was significantly elevated in tumor tissue as compared to non-tumorous tissue from the same patients (GEO: GSE14520, probe set 204881_s_at). **(B)**, a comparison between HCC and normal donor liver tissues resulted also in an increase of *UGCG* in tumors (GEO: GSE64041). *T*-test was used to compare *UGCG* expression levels between tumor and normal samples, paired *t*-test to compare tumor and non-tumor samples.

*UGCG* was significantly elevated comparing tumor versus non-tumorous tissue from the same patients in a paired microarray analysis (Figure 1A); n=233, for each group, p<0.001. In a second dataset normal donor liver tissues were compared with HCC. The expression of *UGCG* in tumorous tissue was in this data set again higher than in normal control liver tissue (Figure 1B); healthy-donor, n=5; tumor, n=60; p<0.001. Our results may imply that downregulation of the gene could affect cancer growth.

### Tumor growth in *Ugcg*^f/fAlbCre^ liver is retarded

**Figure 2:**
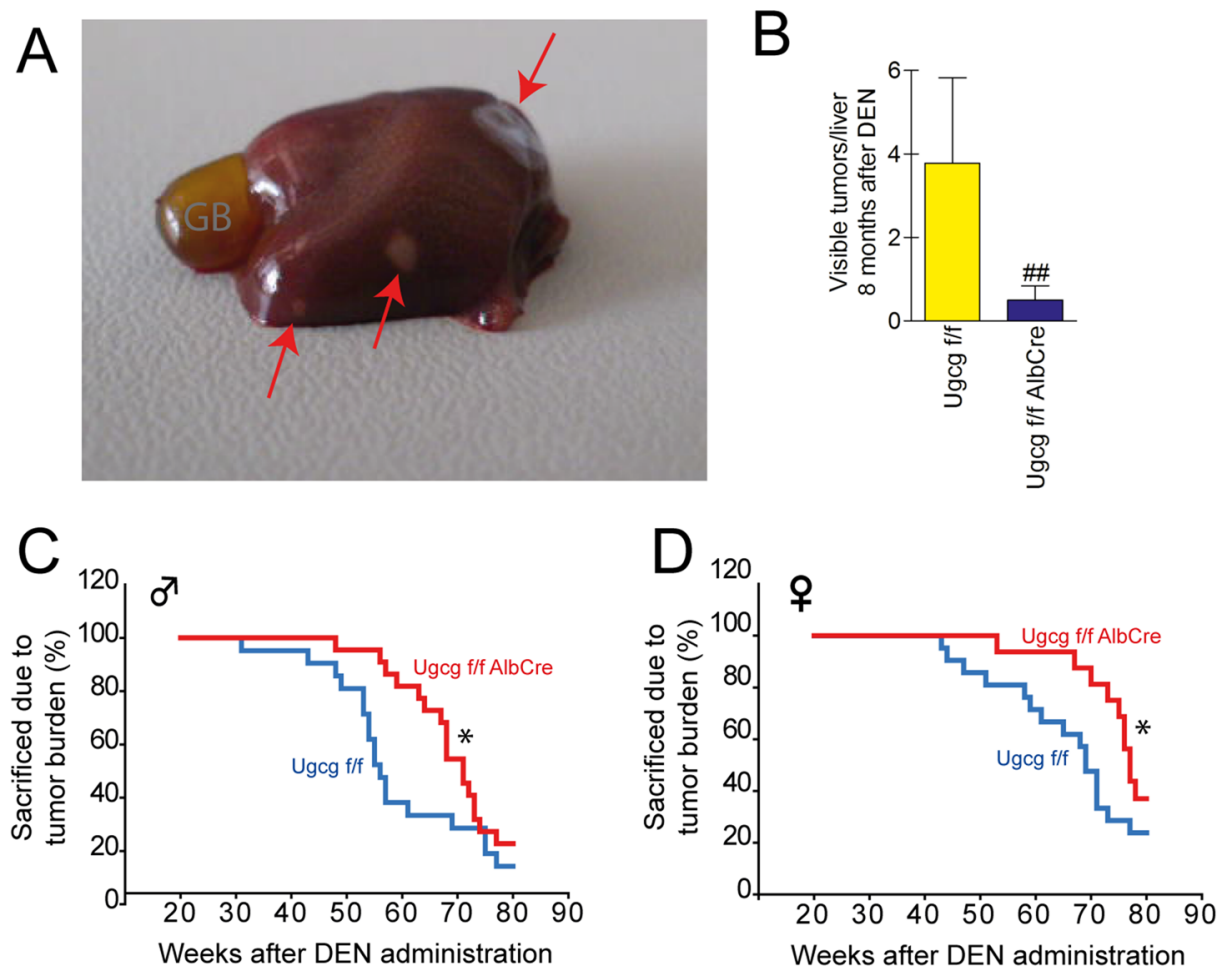
*Ugcg*-deficient mice show delayed DEN-induced life threatening tumor burden **(A)**, 8 months after DEN application small tumors of 1 to 2mm^2^ occurred as indicated by red arrows; GB, gall bladder. **(B)**, lower numbers of macroscopically detectable tumors on the liver surface were seen in GSL-deficient liver (^##^, p<0.0075, Mann-Whitney test). **(C and D)**, *Ugcg*^f/f^ controls suffering from tumor burden in the liver had to be sacrificed earlier than mice with *Ugcg* deficiency; ^*^, p= 0.023 per group and p=0.050 per gender, by Cox-regression; n= 18 to 21 per genotype and gender. The tumor development occurred gender specific ∼15 weeks earlier in males (C) than in females (D).

To investigate whether deletion of *Ugcg* would impact HCC development in mice, an endogenous cancer model was initiated in which *Ugcg* and consequently their synthesis products, *i.e.* glycosphingolipids, were eliminated cell-specifically in hepatocytes. GSL deficiency resulted in a delay of liver tumor initiation. All control mice (n=10) showed 2 to 3 tumors in mean with a diameter of 1-2 mm after 32 weeks (Figure 2A, 2B). In contrast, only 2 of 9 mice with liver specific GSL deficiency developed macroscopically detectable tumors (2 and 3 tumors, respectively) with a size of 1-2mm in diameter. All other *Ugcg*^f/fAlbCre^ mice were macroscopically tumor free after eight months of tumor induction.

We monitored a second group of animals up to 80 weeks after DEN treatment. Palpable tumors in liver of the *Ugcg*^f/f^ control groups of both males and females (Figure 2C and 2D) occurred earlier than in *Ugcg*-deficient animals. More than 50% of control males had to be sacrificed due to critical tumor load already 55 weeks after DEN treatment (Figure 2C). In contrast more than half of male *Ugcg*-deficient mice lived approximately 70 weeks before they were seen with macroscopic tumor load. Similar differences were observed with females except that palpable tumor growth occurred later than in males (Figure 2D).

### Numbers and size of hepatocellular dysplastic foci (AHF) are significantly lower in livers with *Ugcg* deficiency

**Figure 3:**
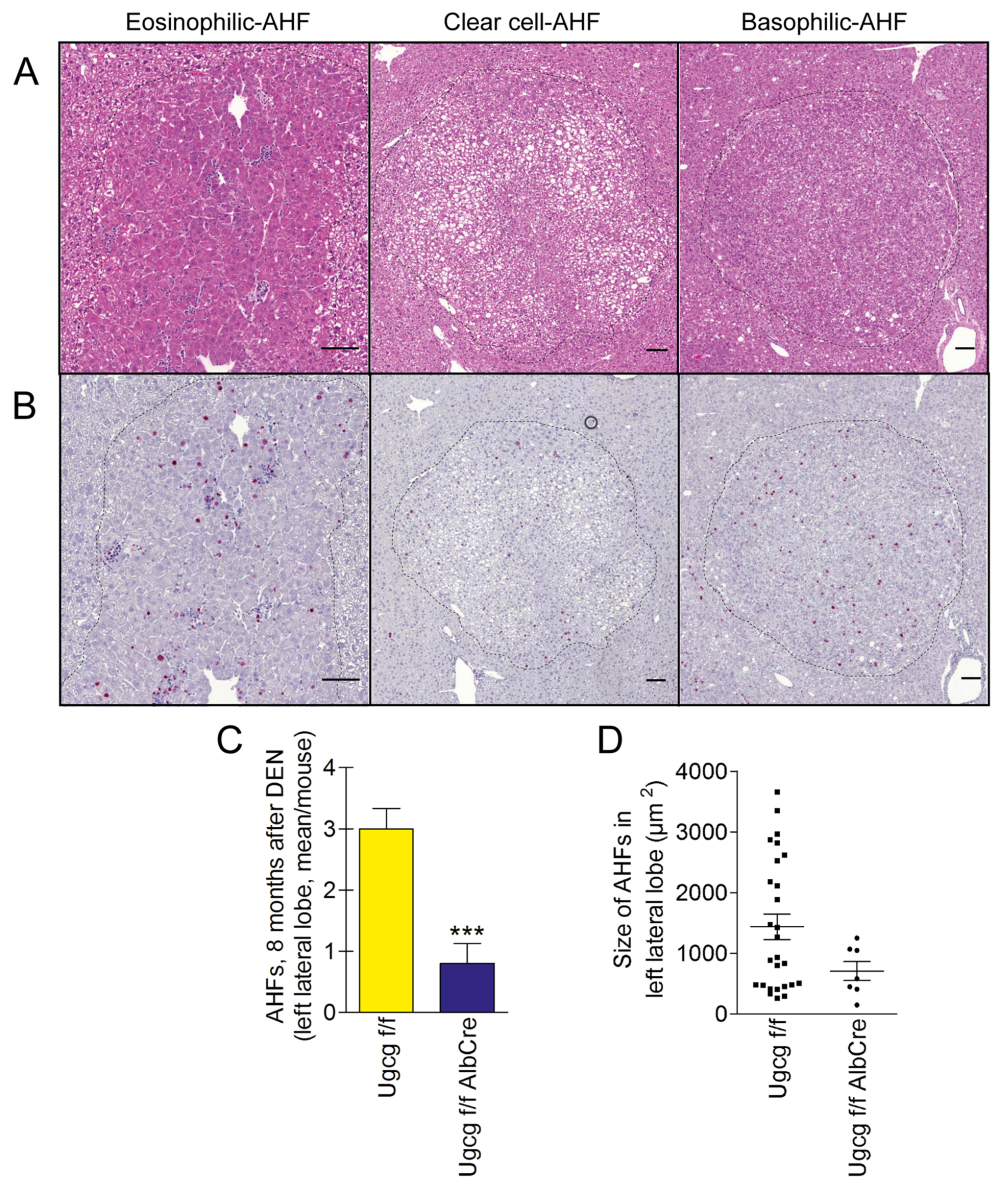
Number and area of dysplastic hepatocellular foci (AHFs) is reduced in *Ugcg*-deficient liver upon DEN treatment **(A and B)**, H&E staining (A) and Ki67 staining (B) of dysplastic foci; scale bars, 100µm. **(C)**, significantly decreased number of AHFs was seen in mutant as compared to control livers. **(D)**, the area of AHFs in cross sections of the left lateral lobe was smaller in *Ugcg*-deficient liver than in control liver (summary of all detected foci; note, differences were not significant (p<0.09) due to the high standard deviation in control group). *Ugcg*^f/f^ controls n=10 and *Ugcg*^f/fAlbCre^ n=9, males. Graphs show mean values ± SEM. Significance, ^***^, p < 0.001 using student’s two-tailed *t*-test.

We dissected the left liver lobe into 7 equal parts to elucidate number, area, and morphology of AHFs eight months after DEN treatment. Histologically, three types of dysplastic foci were identified, eosinophilic-, clear-cell-, and basophilic foci [52] (Figure 3A) with an increase in Ki67-positive cells (Figure 3B). The number of Ki67 positive cells and their distribution within foci were not different comparing control- versus *Ugcg*-depleted mice. Numbers of AHFs, significantly decreased in mutant as compared to control livers (0.80 ± 0.32 vs. 3.0 ± 0.33 per left lateral lobe; p<0.001) (Figure 3C). In addition, AHFs in liver of control mice displayed larger areas as compared to *Ugcg*-depleted livers (1437µm^2^ ± 209 vs. 807 ± 156µm^2^; Figure 3D).

In order to exclude that Ugcg deficient livers would respond more sensitive to DEN-injections than control livers, mice were sacrificed 24h and 96 hours after DEN application and the acute response was elucidated. Neither the area of necrosis nor the numbers of TUNEL-positive-, Ki67-positive-, and F4/80-positive cells, as well as the secretion of the liver enzyme ALT were changed upon Ugcg depletion (Supplementary Figure 1A-1E). Immunohistochemistry of the free radical species marker 3-nitrotyrosine (NTS) as well as the ER-stress marker KDEL did also not show differences comparing Ugcg-deficient- vs. control livers (Supplementary Figure 2). Our data suggested that Ugcg deficiency did not impact the DEN-induced acute liver damage.

### *Ugcg* deficiency induces elevation of binucleation in hepatocytes

**Figure 4:**
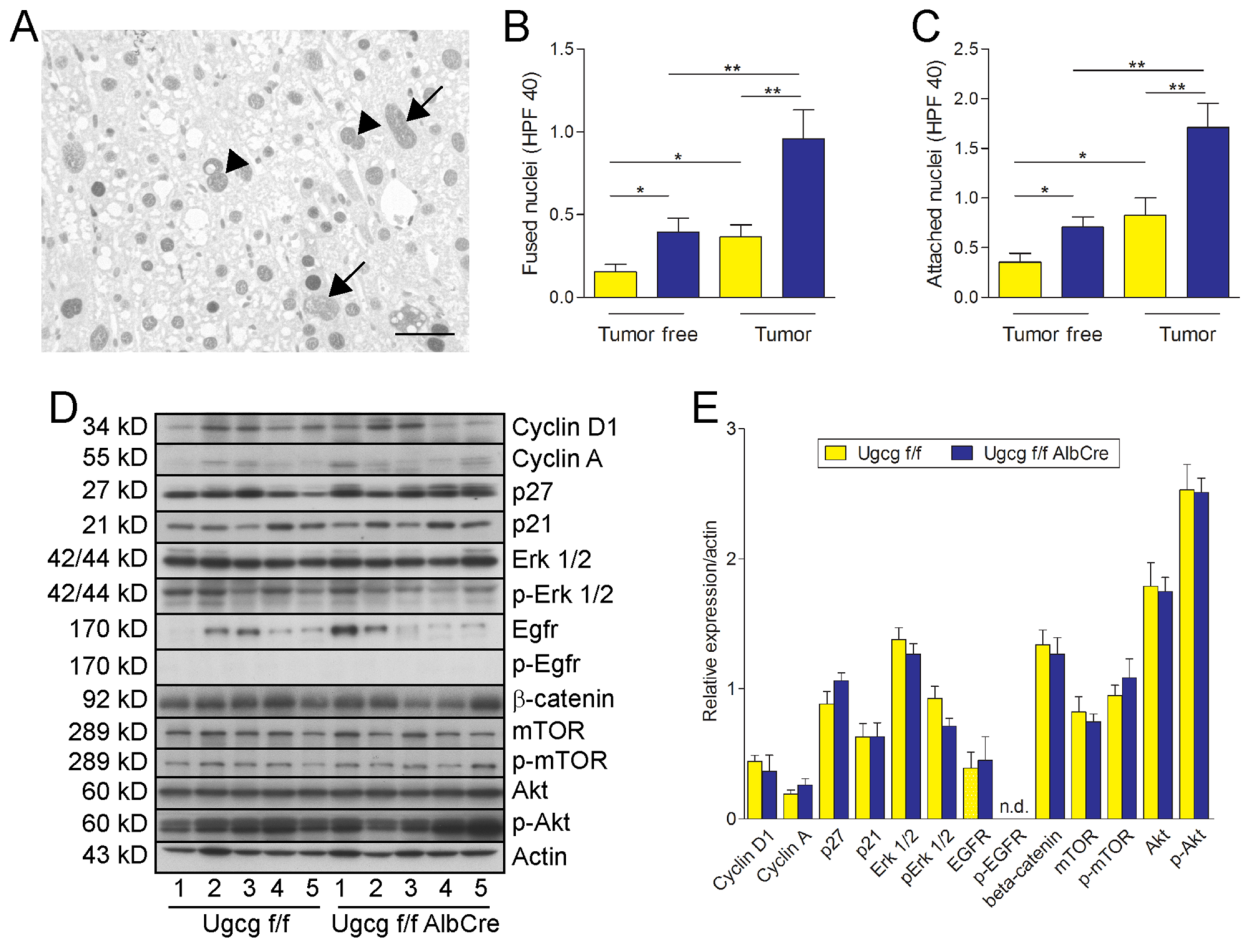
*Ugcg* deficiency causes increased numbers of multinucleated cells; cell cycle protein expression is not affected **(A)**, Ki67 staining of tumor liver of control mice. Arrows label fused nuclei and arrowheads attached nuclei. **(B and C)**, *Ugcg*-depleted liver and corresponding tumors showed increased numbers of bi-(multi) nucleated cells as compared to controls. Both the numbers of fused nuclei (B) and attached nuclei (C) were significantly elevated in *Ugcg*-deficient tissue; n=13 to 14 per group, males; graphs show mean values ± SEM. Significances, ^*^, p < 0.05; ^**^, p < 0.01 using student’s two-tailed *t*-test. **(D and E)**, the expression of proteins potentially involved in cell cycle regulation was not significantly altered in *Ugcg*-deficient tumor tissue although a slight increase of tumor suppressor p27 (p=0.15) and decreased expression of p-Erk 1,2 (p=0.09; n=5, each, males) was observed (D, western blot analysis; E, quantification); note the heterogeneity of the tumor samples.

As an indicator for impaired cytokinesis in Ki67 positive hepatocytes both the number of cells with fused nuclei and with closely attached nuclei significantly increased in *Ugcg*-deficient normal liver and particularly in tumors (Figure 4A-4C).

The expression of proteins involved in cell cycle, such as cyclins and the MAP-kinase Erk were determined. Although there was a tendency for decreased Erk and p-Erk and elevated tumor suppressor p27 in *Ugcg*-depleted tumors, none of these proteins was significantly changed comparing tumor tissue of control- with *Ugcg* deficient liver tumors (Figure 4D and 4E, quantification). The mTOR/Akt signaling pathway was also not affected in *Ugcg* deficient liver tumors; there was a relevant variation in protein expression reflecting the heterogeneity of tumors.

### *Ugcg*-deficient mice lack hepatocyte-typical GSLs

**Figure 5:**
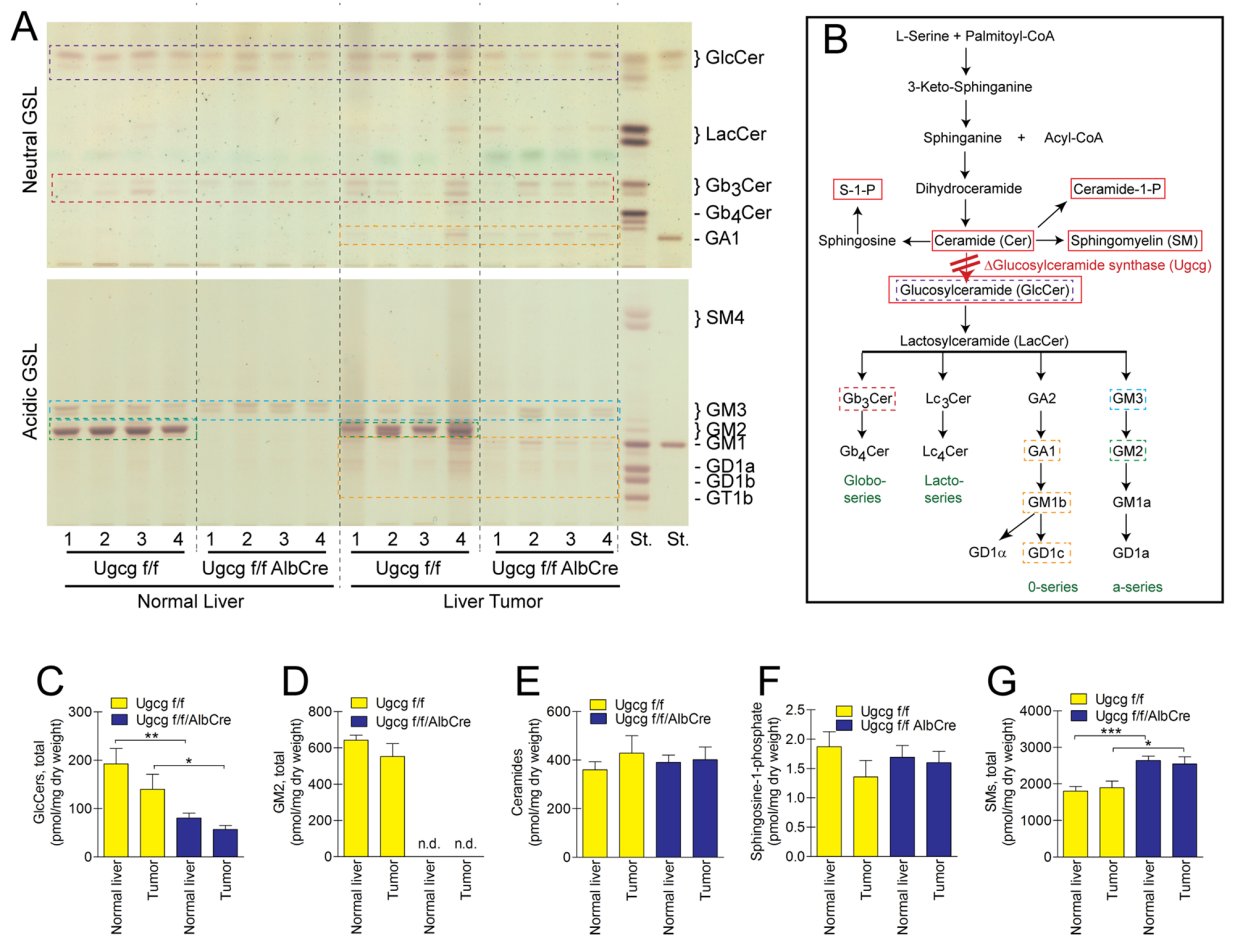
*Ugcg* deficiency results in deletion of GSLs in normal liver- and respective tumor tissue **(A)**, TLC of four normal- and corresponding tumor tissue samples of control and *Ugcg*-deficient liver. Tumor samples showed a slight variability of their GSL constitution. Some minor occurring bands (orange dotted boxes in liver tumor samples) could be verified belonging to the 0-series GSLs likely derived from liver infiltrating immune cells (see also Supplementary Figure 3). For designation of other liver-specific GSLs, compare dotted boxes in (A) with (B). **(B)**, sphingolipid synthesis pathway. Sphingolipids measured by mass spectrometry are indicated by red boxes. **(C and D)**, both glucosylceramide and GM2 were slightly but not significantly less synthesized in tumor tissue of control liver. Hepatocyte-specific ganglioside GM2 was absent in *Ugcg*-depleted normal liver and tumor (D); n.d., not detectable. Lipid extracts were further analyzed by mass spectrometry to investigate whether *Ugcg* deficiency would have caused a switch to other sphingolipid species than GSLs. **(E and F)**, ceramides (E) which were described to have pro-apoptotic- (anti-cancer) and sphingosine-1-phosphate (F) with pro-proliferative properties (pro-cancer) were not essentially changed neither in tumor of *Ugcg*^f/f^ liver nor in normal and tumor of GSL-depleted liver. **(G)** Only sphingomyelin was increased in the same molar range as GSLs decreased; n=8 per group, males; shown are mean values ± SEM. Significances, ^*^, p < 0.05; ^**^, p < 0.01; ^***^, p < 0.001 using student’s two-tailed *t*-test. For more detailed analysis, see Supplementary Figure 4.

We extracted GSLs from normal liver and respective tumor tissue and analyzed for tumor-specific GSLs. Hepatocytes from normal liver of wildtype mice contained almost exclusively GlcCer and GM2 (Figure 5A) [48]. In *Ugcg*^f/fAlbCre^ normal liver and tumor, the hepatocyte-specific ganglioside GM2 was completely absent (Figure 5A). GM3 appeared almost unchanged. This GSL is not present in isolated wildtype hepatocytes and thus GM3 together with residual amounts of GlcCer in total liver extracts can be attributed to cells, not targeted by the albumin-cre mediated *Ugcg* deletion such as endothelial-, Kupffer-, and T cells [48]. GlcCer and GM2 tended to have less steady state levels in tumor- as compared to normal liver in control animals (Figure 5C, 5D). In some of the liver tumor extracts additional minor bands have been detected in the acidic fraction of both control and *Ugcg*-deficient mice. The composition and migration of these compounds correlated with the pattern found in murine macrophages/DCs and or T cells which express predominantly the 0-series GSL derivatives GA1, GM1b, GD1α or GD1c [53, 54] (Figure 5A, 5B). To confirm the presence of these compounds, neutral GSLs were stained by immuno-TLC for presence of GA1 (Supplementary Figure 3A/3B). Also the neutral products from acidic GSLs after *vibrio cholereae* sialidase treatment on TLC and consecutive anti-GA1 staining resulted in a similar pattern as the one obtained from bone marrow derived dendritic cells/macrophages (Supplementary Figure 3C). One may therefore assume that those “tumor associated“ GSLs have been synthesized by immune cells.

### *Ugcg* deficiency in livers leads to an increase of sphingomyelin

We quantified sphingolipids by LC-MS/MS to investigate the effect of *Ugcg* deficiency on sphingolipid synthesis pathways. Differences could not be detected in the ceramide- (Figure 5E, Supplementary Figure 4B) and sphingosine-1-phosphate (S1P) concentration (Figure 5F) of normal- and tumor livers of *Ugcg*^f/fAlbCre^ mice as compared to the respective control tissue. Ceramide-1-phosphate expression was below the detection limit in all investigated samples. Only sphingomyelin increased in *Ugcg*^f/fAlbCre^ normal liver and tumor (Figure 5G; Supplementary Figure 4C) to a similar extent in molar quantities as GSLs decreased (Figure 5C/5D; Supplementary Figure 4A) highlighting a compensatory interaction between the GSL- and the SM-synthesis pathway in hepatocytes and avoiding a suicidal increase in cellular ceramide concentrations.

### Numbers of F4/80 and CD3 positive immune cells are unaltered in GSL-deficient liver

**Figure 6:**
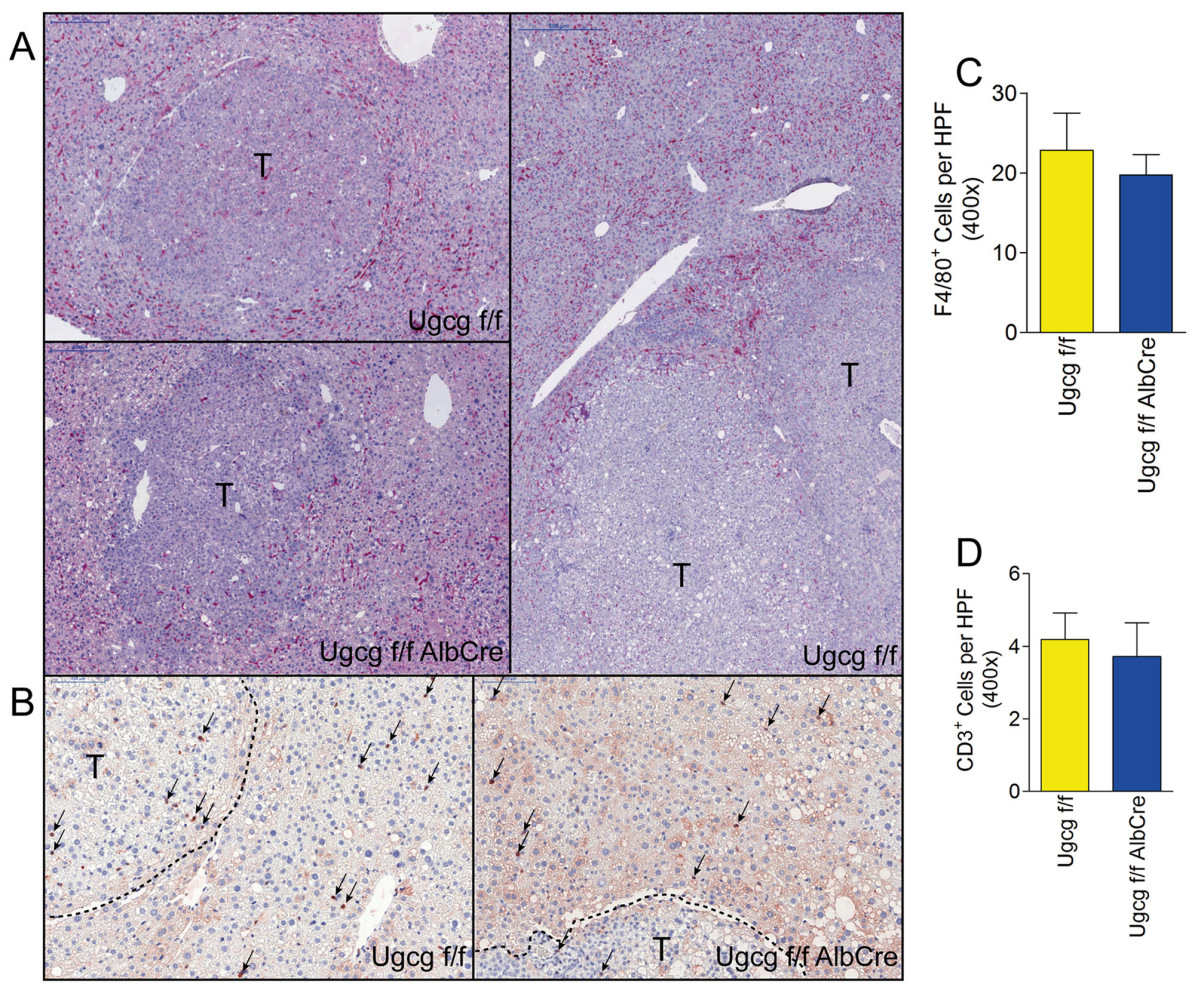
Numbers of F4/80 and CD3 positive immune cells are similar in control- and *Ugcg*^f/fAlbCre^ liver **(A)**, F4/80^+^ cells (*e.g.* macrophages and Kupffer cells) were numerous at the edges of tumors (T). **(B)**, CD3^+^ T cells (arrows) were much less in number than F4/80^+^ cells; scale bars, 100µm. **(C and D)**, no differences in F4/80^+^- (C) and CD3^+^ (D) cell-numbers were detectable in *Ugcg*^f/f^ vs. *Ugcg*^f/fAlbCre^ liver ; n=13 to 14 per group, males.

F4/80-positive cells (macrophages and Kupffer cells) accumulated at the tumor edges in *Ugcg*^f/f^ control- and *Ugcg*^f/fAlbCre^ liver (Figure 6A). Tumors itself were essentially devoid of F4/80-positive cells. CD3-positive T cells were much less in number than macrophages/Kupffer cells and similar in both groups (Figure 6B). Also, the total number of F4/80^+^ and CD3^+^ cells in normal liver were not significantly changed in control- as compared to *Ugcg*-deficient mice (Figure 6C/6D).

### Proliferation of hepatoma cells and growth of cell microspheres in culture decreases significantly upon *Ugcg* blockade

**Figure 7:**
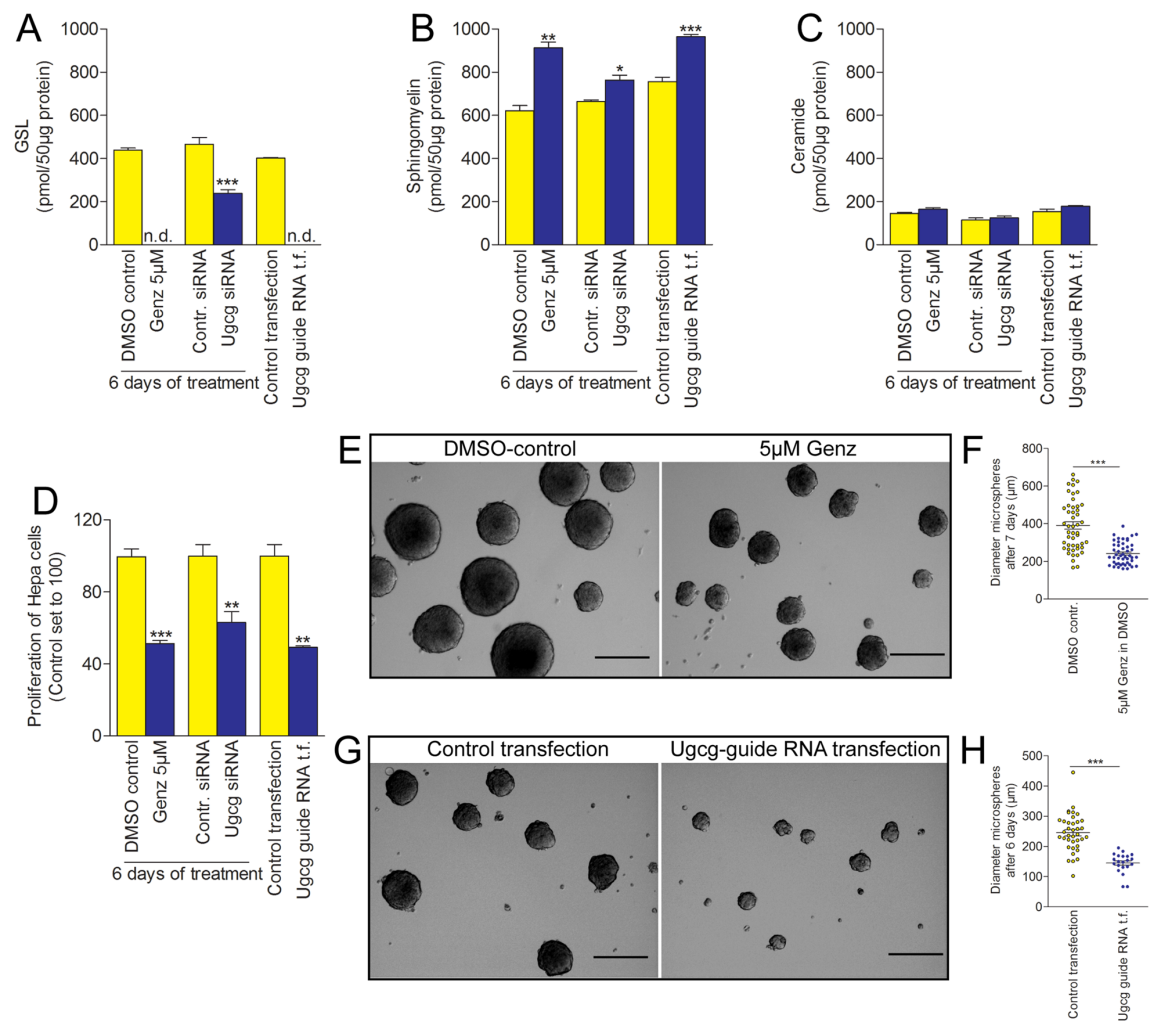
Proliferation of hepatoma cells and hepatoma microspheres is drastically reduced upon *Ugcg* repression **(A)**, the GSL synthesis in hepa cells either treated with *Ugcg* inhibitor Genz 123346 or transfected with *Ugcg* guide RNA was almost completely inhibited. *Ugcg* siRNA transfection resulted in ∼50% decreased expression of GSLs (A, Supplementary Figure 5). **(B)**, the decrease in GSLs was equalized by an approximately equimolar increase of sphingomyelin. **(C)**, ceramide expression was principally low in these cells and not affected by the *Ugcg* depletion. **(D)**, the proliferation of hepatoma cells significantly decreased upon GSL depletion. Shown are the results from one representative experiment out of at least three independent experiments, each; n=3 per group with similar results. **(E-H)**, tumor-like microspheres cultivated from Hepa1-6 cells treated either with 5µM Genz (E and F, quantification) or transfected with *Ugcg* guide RNA (G and H, quantification) had a smaller diameter as control microspheres; graphs represent mean values ± SEM. Significances, ^*^, p < 0.05; ^**^, p < 0.01; ^***^, p < 0.001 using student’s two-tailed *t*-test, respectively; scale bars, 100µm.

In order to place the *in vivo* findings into a cell biologic context, *Ugcg*-repressed Hepa 1-6 hepatoma cells were analyzed with regard to proliferation, morphology, and the growth of tumor microspheres. GSL synthesis in Hepa 1-6 cells was blocked by different approaches either with Genz 123346 or Miglustat (N-butyldeoxynojirimycin, NB-DNJ) both specific and potent inhibitors of the glucosylceramide synthase, with *Ugcg* siRNA or with *Ugcg* guide RNA applying the CRISPR/Cas system. Silencing of *Ugcg* by 1µM or 5µM Genz for 6 days or guide RNA transfection resulted in a drastic decrease of GSL expression (Figure 7A; Supplementary Figure 5A, 5B, 6C). Marked reduction of GSL expression by ≥50% was achieved upon *Ugcg* siRNA treatment for 6 days (Figure 7A; Supplementary Figure 5C) and by addition of Miglustat to the culture medium (Supplementary Figure 6E). Analogous to the data in liver of *Ugcg*^f/fAlbCre^ mice, SM was elevated to an approximately equimolar extent as GSLs decreased (Figure 7B). Ceramide levels remained unchanged upon *Ugcg* silencing (Figure 7C). Downregulation of *Ugcg* did not change the structural integrity of Hepa 1-6 cells (Supplementary Figure 7A, 7B), as well as their mitochondrial membrane potential (Supplementary Figure 7C).

We have observed significantly reduced proliferation by all ways of *Ugcg* silencing (Figure 7D; Supplementary Figure 6A and 6D). The strongest restriction in proliferation was achieved with 5µM Genz and Ugcg guide RNA (Figure 7D). A lower dose of Genz (Supplementary Figure 6A) as well as Ugcg siRNA (Figure 7D) and Miglustat (Supplementary Figure 6D) still resulted in significant reduced proliferation but were less effective.

Both Genz treatment and *Ugcg* guide RNA transfection significantly reduced growth of Hepa 1-6-derived tumor cell microspheres. Genz-treated microspheres displayed a mean diameter of 241.7µm ± 8.5µm as compared to DMSO control microspheres (389.7µm ± 19.3µm; Figure 7E and 7F, quantification; p<0.001) after 7 days. *Ugcg* guide RNA transfected microspheres developed a mean diameter of 144.9µm ± 7.3µm as compared to control microspheres (245.5 µm ± 10.3µm; Figure 7G and 7H, quantification; p<0.001) after 6 days of culture.

To investigate whether the proliferation of other hepatocellular carcinoma cells than Hepa 1-6 cells would be affected upon *Ugcg* silencing, human hepatocellular carcinoma derived HepG2 cells were treated with 1µM and 5µM Genz. The proliferation of HepG2 cells decreased also significantly (Supplementary Figure 6B) suggesting that *Ugcg* silencing indeed has a critical impact on cancer cell growth.

### Hepatoma cells show delayed cytokinesis after *Ugcg* repression

**Figure 8:**
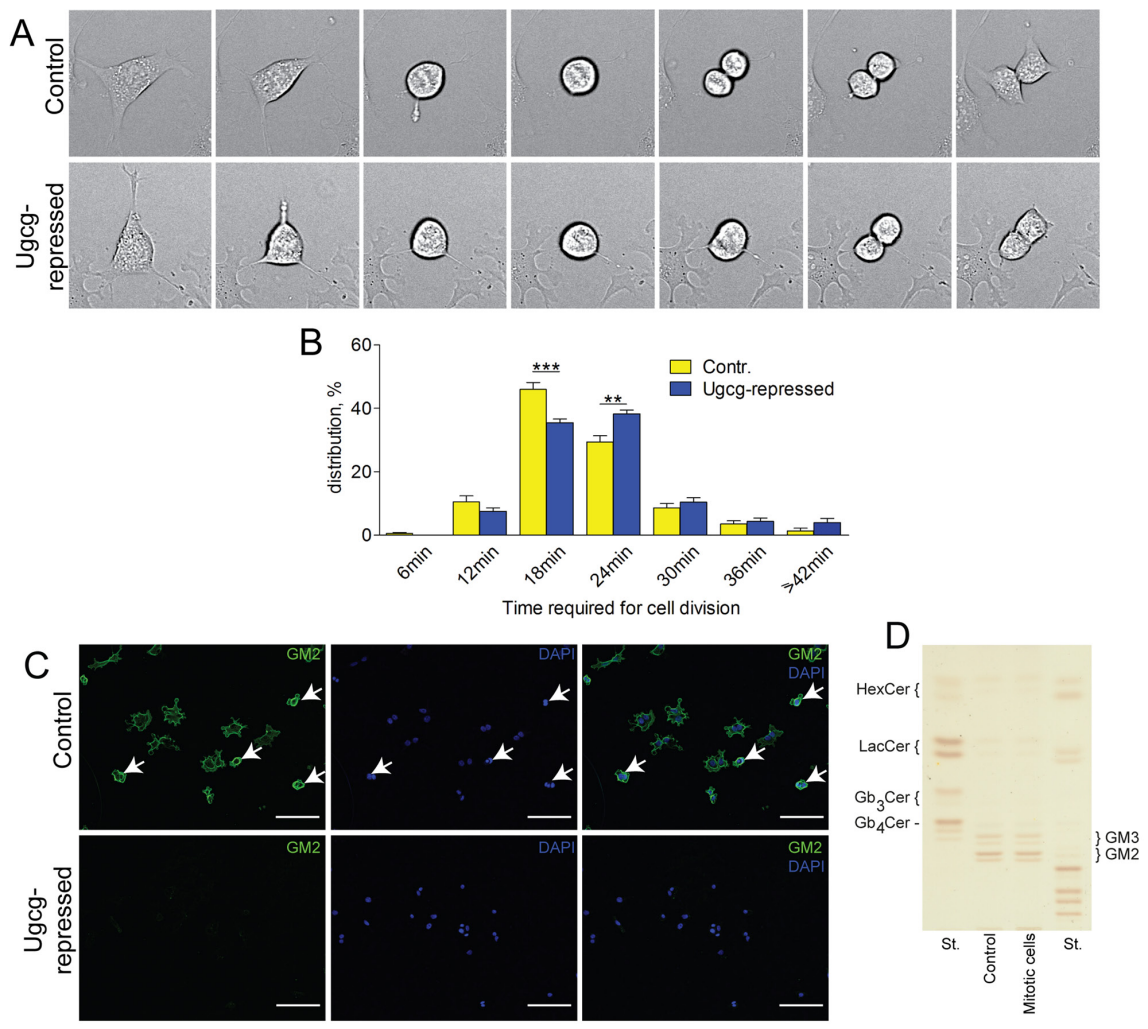
*Ugcg* silencing retards cytokinesis **(A and B)**, control and *Ugcg*-repressed Hepa 1-6 cells were monitored by live cell imaging for 24h. (A), *Ugcg* siRNA-treated cells needed more time for cell division than controls (images were taken every 6 minutes). (B), a majority of the control cells (46%) needed 18min to complete cell division, whereas *Ugcg* repression leads to a delay of cytokinesis and only 35.5% of treated cells completed cell division after 18min; n= 10 power fields with ∼ 30 cell divisions, each; shown are mean values ± SEM. Significances, ^**^, p < 0.01; ^***^, p < 0.001 using student’s two-tailed *t*-test. (**C**, upper panel), staining of GM2, the major GSL of Hepa cells, was moderate in rested cells, but showed a compact expression at the plasma membrane in dividing, rounded cells (mitotic cells are indicated by arrows). **(D)**, an overall increase of GSLs in mitotic Hepa cells was not observed. (C, lower panel), GSL synthesis was significantly reduced by Genz treatment of the cells for 5 days; scale bars, 100µm.

By live cell imaging, we detected a delay in cell division of Hepa cells (Figure 8A and 8B, quantification). Almost half of the control cells finished cytokinesis within 18min (46.0% ± 2.2%) but only one third of the cells upon *Ugcg* silencing (35.5% ± 1.2%; p<0.001). A significant portion of *Ugcg*-repressed cells (38.3% ± 1.2%) needed 24min for finishing cytokinesis vs. 29.4% ± 1.9% of the control cells; p<0.01 (Figure 8B). When cells entered the G2-/M-phase of the cell cycle, GM2 staining increased significantly at the plasma membrane (Figure 8C), although the total GSL content in mitotic hepatoma cells did not change (Figure 8D).

GM2 staining could not be detected in the area where the mitotic spindle formed (Supplementary Figure 8A, inset). Both microtubule spindle formation and the actin cytoskeleton were not altered upon *Ugcg* depletion (Supplementary Figure 8A lower panel, Supplementary Figure 8B). Recycling endosomes, as indicated by Rab11 immunofluorescence, stained predominantly in the cytosol of both control and *Ugcg*-depleted cells (Supplementary Figure 8C) and consequently did not overlap with GSL expression which was predominantly restricted to the plasma membrane (Supplementary Figure 8C upper panel). Thus, GM2 did not seem to be involved in trafficking of recycling endosomes.

## DISCUSSION

To investigate GSL-dependent effects on liver carcinogenesis, we induced liver tumors by application of DEN in mice with hepatocyte-specific genetic deletion of GCS [48]. These animals appeared particularly suitable to be used for studying cancer development in the absence of GSLs as they do not show any spontaneous phenotype [48]. Although other cancer models are available, in the present study we explicitly concentrated our focus on the DEN-model as epidemiological studies imply that nitrosamines may cause cancer [55]. DEN bioactivation due to its alkylation by cytochrome P450 induces DNA adducts which affect DNA replication [56] and trigger cancerogenic pathways, particularly in the liver.

Hepatocellular GSL deficiency did not prevent tumor development. However, we observed a critical impact of GCS expression as the development of endogenously induced hepatocellular carcinoma was significantly delayed under *Ugcg* deficiency. Since the expression of *UGCG* in human HCC is significantly upregulated, our data may have a direct translational impact to human HCC genesis and progression.

A disruption of GSL synthesis influences the flux in sphingolipid synthesis pathways. Increased expression of the GlcCer progenitor ceramide might have been expected; its accumulation has been associated with increased apoptosis [57]. However, ceramide levels were elevated neither in GSL-depleted liver nor in *Ugcg*-repressed hepatoma cells. As ultrastructural changes of organelles and alterations in mitochondrial function of Ugcg-repressed hepatoma cells could not be detected it is not probable that subcellular compartments had elevated ceramide concentrations. Instead, sphingomyelin increased to approximately the same molar extent as GSLs decreased. Further sphingolipid analysis revealed that sphingosine-1-phosphate, described as crucial for cell survival and migration [58, 59], similar as in human HCC [60], was not elevated but rather slightly downregulated in tumors of control mice; it consequently did not appear to contribute to the hepatocellular tumor development and progression.

A critical impact of immune cells could be excluded with regard to cellular tumor rejection in *Ugcg*^f/fAlbCre^ mice. F4/80- and CD3 positive cells showed similar numbers in the periphery of tumors in both control- and GSL-depleted liver. In human and rodent livers binuclear hepatocytes can be observed [61-63] arising from cytokinesis failure [63]. Bi- and polynucleation increased significantly in non-tumorous tissue and even more pronounced in tumors of *Ugcg*-depleted livers as compared to corresponding controls. This result pointed to impaired cytokinesis. A secondary effect due to cell cycle dysregulation did not seem probable as proteins involved in cell cycle were not significantly changed.

*In vitro*, we have also demonstrated that the proliferation both of Hepa 1-6 and HepG2 hepatocellular carcinoma cells was reduced after *Ugcg* depletion. In concordance with the *in vivo* data, tumor microsphere growth was significantly downregulated. Time lapse imaging of Hepa cells revealed increased duration of cell division. The differences are primarily small but accumulate over time.

Staining of GM2 revealed a dense and compact expression at the plasma membrane between G2- and M-phase, starting from the time cells round up until the end of cytokinesis and abscission. A switch of the sphingolipid composition of hepatoma cells from GSLs, mainly GM2, to increased SM synthesis due to repression of *Ugcg*, depleted negative surface charge and likely affected membrane polarity and fluidity. It may thus be surmised that mechanical properties of the cell membrane changed, leading to delayed cytokinesis and increased binucleation of hepatocytes. Our results corroborate data from Nomura et al. [64] showing that *Ugcg*-deficient *C. elegans* embryos developed multinucleated cells causing decreased brood size of the animals. Also Atilla-Gokcumen et al. demonstrated that alterations of the lipid content in HeLa S3 cells, achieved by siRNA treatments of synthesizing enzymes, changed their physical properties and caused cytokinesis failure [65].

Secretory transport vesicles either derived from recycling endosomes or by abscission from the Golgi apparatus are indispensable for the transport of lipids and proteins along microtubules to the intracellular bridge. Here they supply their content for the generation of new membrane compartments during late steps of cytokinesis [66]. Whether GSLs are involved in trafficking of secretory vesicles and whether repression of synthesis of GSLs retarded the transport of those vesicles cannot be definitively dissected due to the lack of appropriate analysis techniques for endogenous lipids. Up to now it appears impossible to label GSLs in such a way that neither their structure and size nor their polarity is affected. GM2 staining however did largely not overlap with the recycling endosomal marker Rab11. An involvement in cellular recycling processes might therefore be excluded.

In the present study we have shown for the first time that genetic deletion of *Ugcg* restricts tumor growth in endogenous diethylnitrosamine-induced hepatocellular carcinoma due to delay and failure of cytokinesis. *Ugcg* repression, whether genetically induced or chemically achieved by GCS inhibitors which are already used clinically to treat GSL storage diseases, might be a promising approach for the treatment of hepatocellular carcinoma.

## MATERIALS AND METHODS

### *UGCG*-gene expression analysis

Two publicly available GEO HCC datasets with Affymetrix microarray measurements GSE14520 [49, 50] and GSE64041 [51] were used. Normalized and log2-transformed expression values of tumor, non-tumor and healthy donor samples were analyzed. *T*-test was used to compare *UGCG* expression levels between tumor and normal samples, paired *t*-test to compare tumor and non-tumor samples. P-values below 0.05 were considered statistically significant. Analysis was performed with software R 3.4.

### Animals

Mice with targeted mutation of the glucosylceramide synthase gene (*Ugcg*) specifically in hepatocytes (*Ugcg*^f/fAlbCre^) [48] as well as control litters (*Ugcg*^f/f^) with congenial C57Bl6 background were used to study the effects of GSLs on endogenously induced liver cancer. All animal experiments were approved by federal law.

### Diethylnitrosamine (DEN) treatment of mice

For cancer induction, mice received a single dose of DEN (25mg/kg, Sigma, Munich, Germany) postnatally at day 15 [67]. Application of DEN at this early time point directs cancer development predominantly to the liver. One group of mice was sacrificed 8 months after DEN application in order to investigate cancer development. Numbers of macroscopically detectable liver tumors were counted. In addition, the left lateral liver lobe was sliced into 7 equal pieces which were embedded in paraffin.

In a long term experiment, a second group was kept for up to 80 weeks.

Mice were sacrificed according to federal animal law when animals started to show palpable tumors and tumor symptoms indicated by reduced food/water uptake and accompanied weight loss, apathy, or abnormal posture. Tumors from those mice were collected for analysis. Acute liver damage in mice was induced by singular i.p. injection of 100mg DEN/kg [67].

### Histology and immunohistology

Sections were stained with hematoxylin/eosin (H&E, Chroma, Köngen, Germany) or anti-Ki67 (DAKO, Hamburg, Germany) [68] to visualize altered hepatocellular foci (AHF). Multinucleation in Ki67 positive cells of normal liver and tumors was determined by counting 10 high power fields at a magnification of 40 in hot spots. Details of anti-nitrotyrosine and anti-KDEL immunohistochemistry have been described in Supplementary Methods.

### Sphingolipid extraction, mass spectrometry, and sialidase treatment of acidic GSL

One part of liver and tumor corresponding to ∼50mg dry tissue has been extracted and analyzed for lipids as described [68]. For further details see, Supplementary Methods.

### Cultivation and transfection of Hepa 1-6 cells with small interfering RNAs (siRNAs)

Hepa 1-6 primary mouse hepatoma cells (ATCC, Manassas, VA, USA) were cultivated in DMEM with high glucose and glutamine, 10% FCS, Pen/Strep, and Hepes (Life Technologies, Darmstadt, Germany). Cells were transfected twice with a mix containing of 1ml DMEM w/o FCS, 20µl Hiperfect (Qiagen, Hilden, Germany), and 6.3µl specific siRNA within 6 days as described in Supplementary Methods.

### GCS inhibition with Genz 123346

Hepa 1-6 cells were cultivated as described before. 2x10^5^ cells in 10 ml culture medium in 10cm tissue culture dishes (Greiner Bio One, Frickenhausen, Germany) containing either 1µM/5µM Genz in DMSO or DMSO as control were incubated for 3d. Cells were counted, split and treated for another 3d as described above. Cells were then harvested and counted.

### GCS inhibition with miglustat

Miglustat (Tocris, Ellisville, MO, USA) is less effective in GCS inhibition than Genz. Therefore, a higher concentration in the medium (100µM in H_2_O) has been used to decrease GSLs in a significant manner [69]. Hepa 1-6 cells were cultivated in analogy to the Genz treatment.

### GSL depletion in Hepa 1-6 cells using CRISPR/Cas9 technology

A CRISPR/Cas9 expression vector with puromycin and ampicillin resistance (pX459, Addgene, Cambridge, MA, USA) was used for integration of the *Ugcg* guide RNA. Hepa cells were transfected as described in detail in the Supplementary Methods. 4x10^5^ targeted- and control cells each in 10 ml culture medium in 10cm tissue culture dishes (Greiner Bio One) were cultivated for 3 days. Cells were harvested and counted.

### Isolation of mitotic Hepa cells

Hepa cells were harvested by mitotic shake off as described [65].

### Cultivation of HepG2 cells and GCS inhibition with Genz 123346

HepG2 primary human hepatocellular carcinoma cells (ATCC, Manassas, VA, USA) were cultivated in DMEM with high glucose and glutamine, 10% FCS, Pen/Strep, and pyruvate (Life Technologies, Darmstadt, Germany). 2x10^5^ HepG2 cells were cultivated in 10cm culture plates (Greiner Bio One, Frickenhausen, Germany) for 6 days in the presence of 1µM/5µM Genz in DMSO or DMSO as control. Medium with or without Genz was changed on day 2 and day 4. Cells were harvested and counted.

### Western blotting

Proteins were isolated as described [70]. Antibodies and blocking reagents can be found in the Supplementary Table 1. Quantification was performed with Image J.

### Tumor microspheres

Tumor-like microspheres were achieved by cultivating ∼ 4x10^3^ Hepa cells per cm^2^ in uncoated tissue plates (Greiner Bio One) for 6 to 7 days.

### Live cell imaging

Live cell imaging was performed using an Olympus CellR microscope. Images were taken in 10 different fields of *Ugcg*-depleted- and control cells every 6min within 24h. The duration from rounding up of the cells at the prophase of the cell cycle until completion of cytokinesis was evaluated for each field.

### TUNEL assay

TUNEL was performed according to the manufacturer’s instructions of the (In Situ Cell Death Detection Kit, Roche, Mannheim, Germany). Apoptotic cells were counted in 20 high power fields (HPF x400).

### Transmission electron microscopy

Hepa 1-6 cells were fixed with 2.5% glutaric aldehyde in 0.05M cacodylate buffer, dehydrated with an alcohol series and embedded in araldite. Sections of 70nm thickness were cut using an Ultracut UCT (Leica, Wetzlar, Germany) and stained as described [68]. Sections were analyzed with an EM 910 and camera CCDK2 (Zeiss, Oberkochen, Germany).

### Measurement of mitochondrial membrane potentials

Hepatoma cells were treated twice with *Ugcg* siRNA or control siRNA. Mitotracker green and -red 100nM each were added to the medium for 30min. Cells were washed, trypsinized and analyzed on a FACS Calibur (BD, Heidelberg, Germany).

## SUPPLEMENTARY MATERIALS FIGURES AND TABLE



## Abbreviations

AHF: altered hepatocellular focus
DEN: diethylnitrosamine
GCS: glucosylceramide synthase
Genz123346: N-[(1R,2R)-1-(2,3-Dihydrobenzo[b] [1, 4] dioxin-6-yl)-1-hydroxy-3-(pyrrolidin-1-yl)propan-2-yl] nonanamide
GlcCer: glucosylceramide
GM2 ganglioside: (GalNAcβ1-4(NeuAcα2-3)Galβ1-4Glcβ1-1Cer)
GSL: glycosphingolipid
HCC: hepatocellular carcinoma
NTS: 3-nitrotyrosine
SM: sphingomyelin
*Ugcg*/*UGCG*: glucosylceramide synthase gene
