# Supplementary material for: Inhibition of hepatocellular carcinoma growth by blockade of glycosphingolipid synthesis
Source: Oncotarget. 2017 Nov 24;8(65):109201–16. doi: 10.18632/oncotarget.22648 (PMC5752514; doi:10.18632/oncotarget.22648)
Supplement: Supplementary file 1 [file oncotarget-08-109201-s001.pdf]

Inhibition of hepatocellular carcinoma growth by blockade of glycosphingolipid synthesis

SUPPLEMENTARY MATERIALS

Sphingolipid extraction, mass spectrometry, and sialidase treatment of acidic GSL

One part of liver and tumor corresponding to ~50mg dry tissue has been extracted as described [1]. Isolated sphingolipids including GSLs, ceramides, and sphingomyelins were investigated by thin layer chromatography (TLC) and mass spectrometry (MS) [1]. For the detection of 0-series gangliosides (GM1b and GD1α) in tumor tissue, three aliquots of the extracts from representative GSL-deficient and control tissues corresponding to 2mg tissue dry weight were loaded on a TLC plate (Merck, Darmstadt, Germany) together with an extract from bone marrow-derived dendritic cells as a control. The TLC plate was divided into 3 parts. One was stained with orcinol reagent to visualize all GSLs. The remaining plates were fixed with 5% polyisobutylmethacrylate (Sigma, Munich, Germany) in chloroform which was 1:10 diluted in n-hexane. The second plate remained untreated and the third plate was treated with sialidase accordingly as described [2]. Thereafter, the plate was blocked with 1% BSA in PBS for 10min and subsequently washed 4 times with neuraminidase digestion buffer (0.2M sodium acetate, 2mM $CaCl_2$ buffer, pH 5.2). The TLC plate was covered with 0.05u/ml of Vibrio cholera sialidase (Sigma) in digestion buffer for 6h at RT. Sialidase was removed by 4 times washing with PBS/0.05% Tween 20. For the detection of GA1 as cleavage product from acidic GSLs of the 0-series, both untreated- and sialidase treated plates were first blocked with 1% BSA in PBS for 30min. Then the plates were incubated with an affinity purified [3] anti-GA1 antibody (Acris, Herford, Germany) 1:50 diluted in 1% BSA in PBS at 4°C overnight. The plates were washed five times with PBS/0.05% Tween-20 and incubated with secondary 1:500 diluted horseradish peroxidase-linked goat anti-rabbit antibodies (Dianova, Hamburg, Germany) in 1% BSA/PBS for 4h at RT. After washing for five times, positive bands were detected using Sigma Fast BCIP (Sigma).

Immunohistochemistry and immunofluorescence

Macrophages/Kupffer cells were stained with rat anti-F4/80 (Acris), 1:500 and secondary biotinylated anti-rat/streptavidin-AP (Vector, Burlingame, CA, USA), 1:200. T cells were stained with goat anti-CD3 (Santa Cruz Biotech, Heidelberg, Germany), 1:100 and secondary biotinylated anti-goat (Dianova), 1:300 and streptavidin-HRP (Vector), 1:200. For indication of oxidative stress an anti-nitrotyrosine antibody (Millipore, #06-284), 1:80 was used followed by biotin anti-rabbit (Dianova), 1:200 and HRP-streptavidin (Vector), 1:200. ER-stress was documented with an anti-KDEL antibody (Abcam, #12223 [10C3]), 1:50, followed by biotin anti-mouse (Dianova), 1:200 and HRP-streptavidin (Vector), 1:200. GM2 was stained in Hepa 1-6 cells with a polyclonal anti-GM2 antibody raised in mice which has been affinity purified on a GM2-octylsepharose column [3]. $5x10^4$ Genz or Ugcg guide RNA-treated Hepa cells were transferred on top of glass coverslips (Marienfeld, Germany) in 6 well plates. The cells were allowed to attach overnight. An equal volume of 4% PFA in PBS was added to the 6-well plate and Hepa cells prefixed for 10min followed by another fixation step with 4% PFA in PBS for 10min. The coverslips were washed three times with PBS for 5min and permeabilized/blocked with 0.01% triton X-100 (Sigma) in PBS at room temperature for 10min (note, higher triton X-100 concentrations or extended treatment time may lead to a dislocation of glycolipids). Blocking was subsequently performed with 1% fat free milk powder in PBS for 30min at room temperature. For the GM2/tubulin staining, the affinity purified anti-GM2 antibody was 1:50 diluted in 1% milk powder in PBS and placed on top of the coverslips over night at 4°C. After three washing steps each with PBS for 5 min, cells were covered with anti-mouse AF-488 (Life Technologies, Darmstadt, Germany), 1:200 in 1% milk powder in PBS at RT for 1h. Coverslips were washed as before, stained with rabbit anti-tubulin (Cell Signaling, Danvers, MA, USA, #2128), 1:200 in 1% milk powder in PBS at RT for 1h. After washing, secondary anti-rabbit AF 594 (Life Technologies), 1:200 in 1% milk powder in PBS was added to the coverslips respectively at RT for 1h.

For the Rab11/GM2 staining, coverslips were treated as described above and first stained with rabbit anti-Rab11 (Cell Signaling, #5589), 1:20 in 1% milk powder in PBS at RT for 1h. After washing, secondary anti-rabbit AF 488 (Life Technologies), 1:200 in 1% milk powder in PBS was added to the coverslips respectively at RT for 1h. After washing, cells were covered by the affinity purified anti-GM2 antibody as described above over night at 4°C. After 3 washing steps each with PBS for 5 min, cells were covered with anti-mouse AF-594 (Life Technologies), 1:200 in 1% milk powder in PBS at RT for 1h. Coverslips were washed as before and for nuclear staining covered with diluted DAPI solution (0.1µg/ml) in 1% milk powder in PBS for 10 min. After a final washing step with PBS, coverslips were embedded

in fluorescent mounting medium (DAKO). For staining of actin filaments cells were fixed as described above and pretreated with 0.1% triton X-100 at RT for 10min. After blocking with 1% milk powder in PBS for 30min, actin was stained with Phalloidin TxRed (Biotrend, Cologne, Germany), 1:200 in 1% milk powder in PBS at RT for 1h. Coverslips were washed and nuclear staining performed as described before. Images were taken with a Keyence BZ-9000 fluorescent microscope (Neu-Isenburg, Germany).

Cultivation and transfection of Hepa 1-6 cells with small interfering RNAs (siRNAs)

$4x10^5$ Hepa 1-6 primary mouse hepatoma cells (ATCC, Manassas, Virginia, USA) in 4 ml culture medium (DMEM with high glucose and glutamine, 10% FCS, Pen/Strep, and Hepes, all from Life Technologies) were transfected with a mix containing 1ml DMEM w/o FCS, 20µl Hiperfect (Qiagen, Hilden, Germany), and 6.3µl specific siRNA; *Ugcg* siRNA si01461551 (20µM) as well as control siRNA si03650318, 20µM; all from Qiagen) in a 10cm culture dish. 5ml culture medium was added after 24h. Cells were cultivated for further 2d and counted. Cells were split in equal number and retransfected as described before for another 3d. For determination of the proliferation, cells were harvested and counted.

GSL depletion in Hepa 1-6 cells using CRISPR/Cas9 technology; cloning of the Ugcg guide sequence into the CRISPR/Cas9 expression vector

Plasmid pX459 (Addgene, Cambridge, MA, USA) with combined guide RNA/Cas9 expression sequence as well as puromycin and ampicillin resistance has been used for insertion of the Ugcg guide oligos. The vector (5µg) was digested with 10u of the enzyme Bbs I (Thermo Scientific, Waltham, MA, USA) at 37°C for 16h. The DNA was purified using a PCR-purification Kit (Qiagen). The guide oligos used were:

Ugcg guide 1 forward: 5'-GTTCGGCTTCGTGC TCTTCG-3'

and Ugcg guide 1 reverse: 5'-CGAAGAGCACGA AGCCGAAC-3'

as well as Ugcg guide 2 forward: 5'-GAATCAGATG ACAGAGAAAG-3'

and Ugcg guide 2 reverse: 5'-CTTTCTCTGTCA TCTGATTC-3',

including the 5'- and 3'-Bbs I overhangs, respectively, were dissolved at a concentration of 150µM. 25µl of the respective forward and reverse oligonucleotide was mixed and annealed (95°C, 4'; 70°C, 10'; followed by cooling down the mixture in a thermomixer (Eppendorf, Hamburg, Germany) to room temperature). For oligo insertion, ~100ng of the purified expression vector, 10µl (~10µg) of the annealed oligo and 1u of T4-ligase and ligase buffer (Life Technologies) in a total volume of 20µl (ad. ddH_2O) were used. In addition, a control ligation without ligase was performed. Ligations were performed at 16°C for 16h. Due to the excess of oligonucleotides (> 40000 fold, mol/mol), dephosphorylation of the plasmid was not necessary to inhibit vector religation. Subsequently, DH5α E. coli (Life Technologies) were transformed with 5µl of the ligation mixtures according to the manufacturer's protocol and spread on LB agar gel plates with 100µg/ml ampicillin. The plates were incubated at 37°C for 16h. Minipreps of single colonies were performed in 5ml LB medium with 100µg/ml ampicillin at 37°C in a bacteria shaker for 16h. The plasmid DNA was isolated using a plasmid mini kit from Qiagen. Minipreps were checked for correct integration of the Ugcg oligonucleotides by PCR and consecutive sequence analysis.

Transfection of Hepa cells with the *Ugcg* guide construct

$1x10^5$ Hepa cells were seeded in a 6-well plate and transfected with a mixture of vector DNA and lipofectamine (Life Technologies) according to the manufacturer's instructions. The selection of transfected cells with 1µg puromycin/ml culture medium was started 24h later.

REFERENCES

1. Jennemann R, Kaden S, Sandhoff R, Nordstrom V, Wang S, Volz M, Robine S, Amen N, Rothermel U, Wiegandt H, Grone HJ. Glycosphingolipids are essential for intestinal endocytic function. The Journal of biological chemistry. 2012; 287:32598-32616.
2. Saito M, Kasai N, Yu RK. In situ immunological determination of basic carbohydrate structures of gangliosides on thin-layer plates. Analytical biochemistry. 1985; 148:54-58.
3. Jennemann R, Sandhoff R, Grone HJ, Wiegandt H. Human heterophile antibodies recognizing distinct carbohydrate epitopes on basidiolipids from different mushrooms. Immunol Invest. 2001; 30:115-129.
4. Yohe HC, Ye S, Reinhold BB, Reinhold VN. Structural characterization of the disialogangliosides of murine peritoneal macrophages. Glycobiology. 1997; 7:1215-1227.

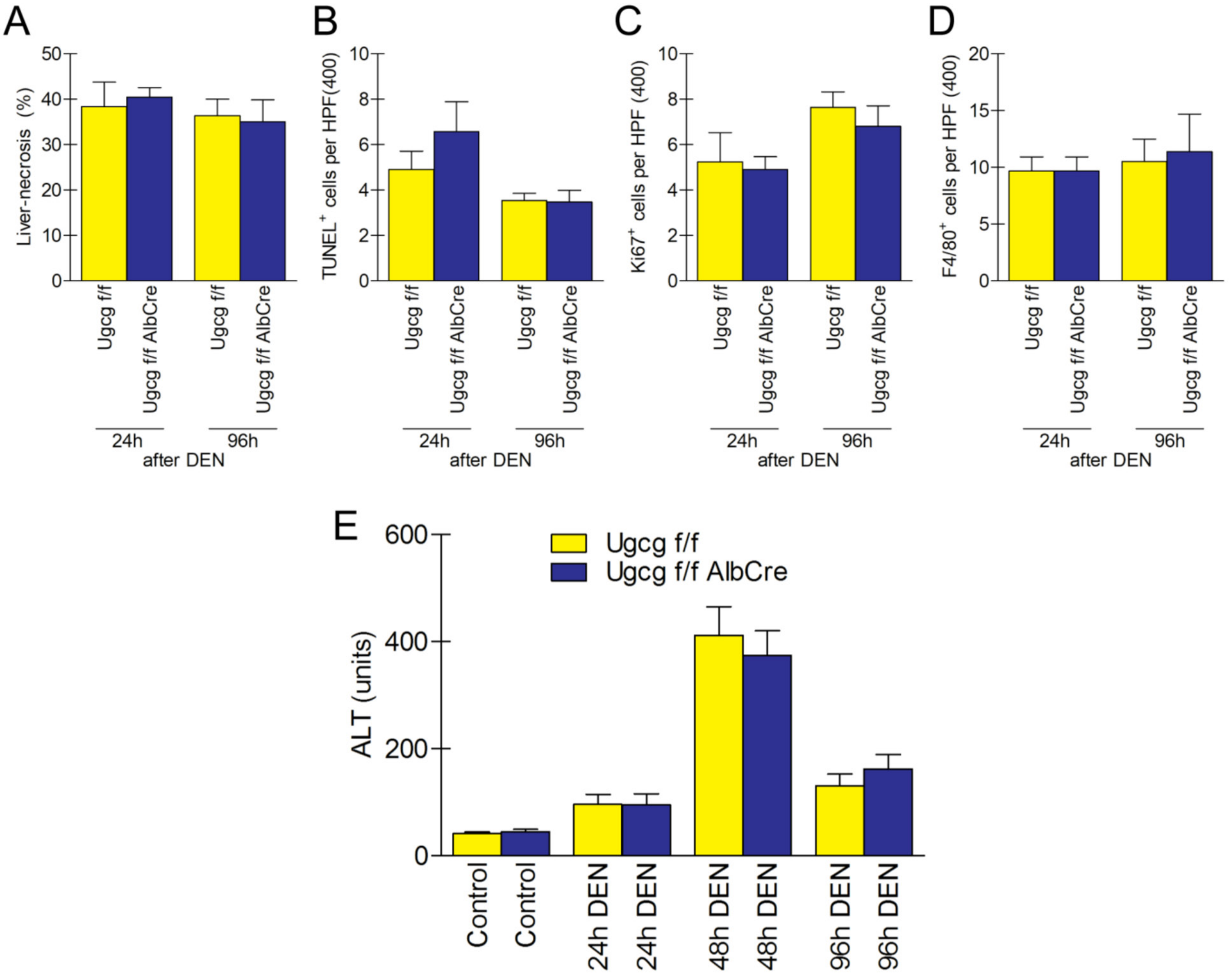

Supplementary Figure 1: DEN-induced acute liver damage was not different in *Ugcg*-deficient- as compared to control livers. (A-D), mice were injected with DEN and sacrificed 24h or 96h later. Liver sections were investigated for necrosis (A), TUNEL-positive cells (B), Ki67-positive cells (C), and F4/80-positive cells. No difference with respect to damage, apoptotic cells, proliferation, and macrophage infiltration was observed between GSL-deficient tissue vs. control liver, neither 24h nor 96h after DEN-injections. **(E)**, the liver enzyme ALT was also not changed. ALT-decrease 96h after DEN-treatment indicated beginning of liver recovery; n=4 to 5 mice per group.

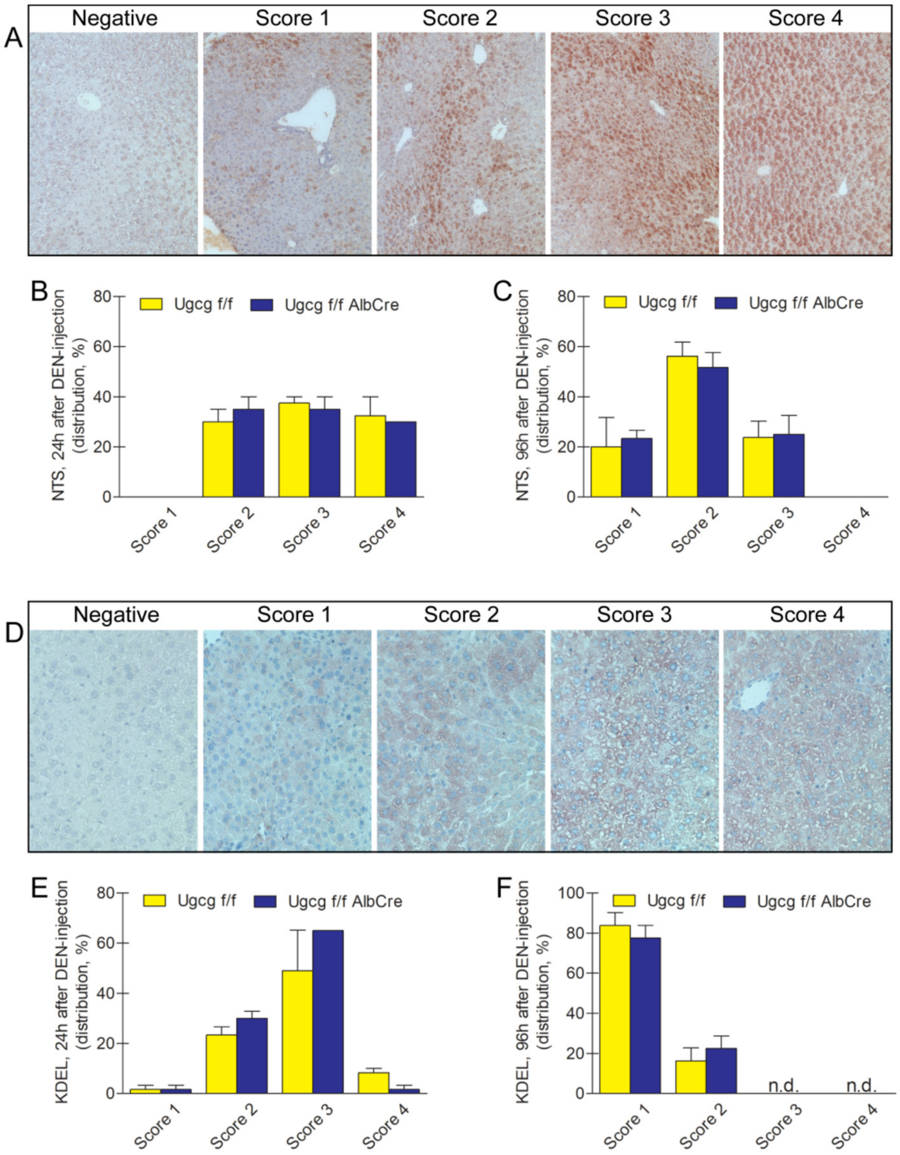

Supplementary Figure 2: 3-Nitrotyrosine (NTS), a marker for free radical species and KDEL, a marker for ER stress are not altered in *Ugcg*-deficient liver after DEN injections. **(A and D)**, immunohistochemistry was categorized according to the grade of NTS- and KDEL-expression into four different scores. **(B and C)**, semi quantitative evaluation of NTS-expression 24h (B) or 96h (C) after DEN-injection. **(E and F)**, semi quantitative evaluation of KDEL-expression 24h (E) or 96h (F) after DEN-injection; 20 high power fields (HPF 40x) were investigated from liver sections of each mouse. DEN application induced a strong NTS- and KDEL-expression after 24h (B and E). NTS- as well as KDEL-expression markedly decreased again 96h after DEN treatment (C and F). Differences were not detected comparing *Ugcg*-deficient- with control animals; n=3 to 4 mice.

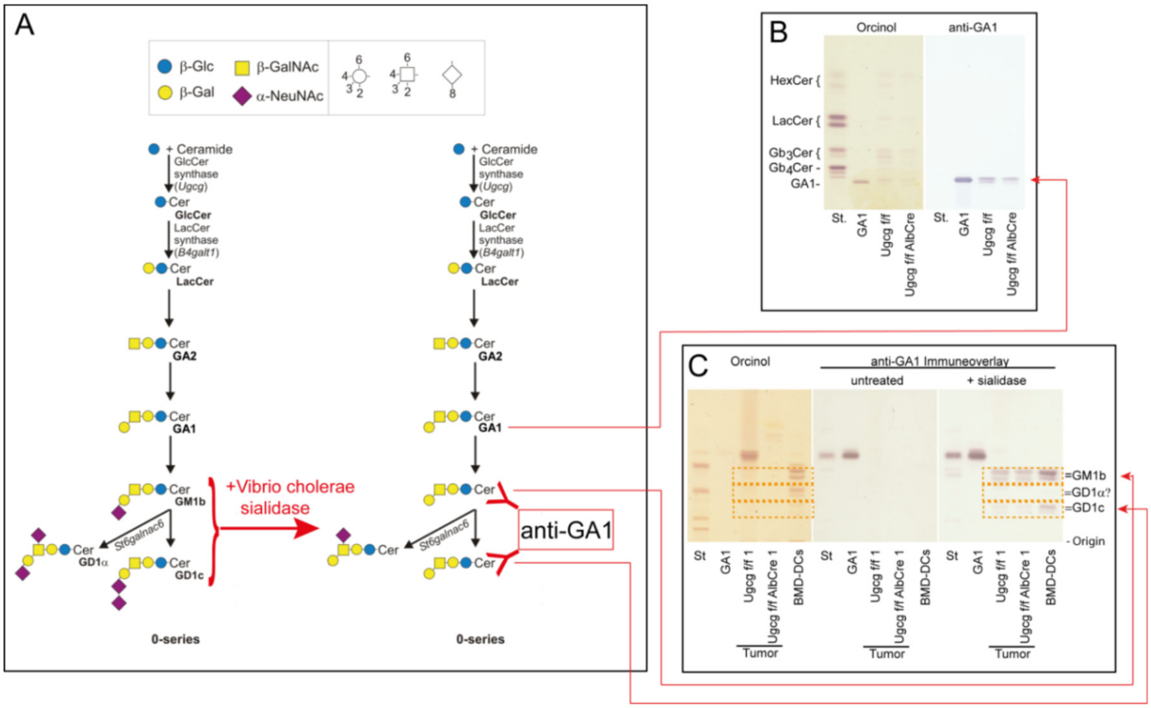

Supplementary Figure 3: GSLs from tumor tissue contain compounds of the 0-series. (A), GA1, also known as asialo GM1 or Gg_4Cer, could be verified in the neutral GSL fraction by anti GA1 immune overlay. GA1 expression was elevated in liver tumors of both *Ugcg*$^{f/f}$- and *Ugcg*$^{f/fAlbCre}$ mice (Figure 4A). **(B)**, gangliosides of the 0-series GSLs are characterized by sialic acid ornamentations on galactosamine and/or on the terminal galactose. Treatment of those GSLs with *vibrio cholerae* sialidase releases the neutral backbone GA1 from the 0-series gangliosides GM1b and GD1c (B). This method distinguishes 0-series gangliosides from a-, b-, and c-series derived ganglio-series gangliosides which cannot be completely cleaved with this sialidase and are negative with anti- GA1 antibodies. Sialidase treatment on the TLC plate and subsequent anti-GA1 staining confirmed the presence of GA1 and consequently the presence of 0-series derived gangliosides in the acidic GSL fraction **(C)**. 0-series gangliosides typically can be found in mouse immune cells including bone marrow-derived DCs/macrophages (BMD-DCs) and T cells [4], suggesting that these compounds in the tumor extracts are derived from liver infiltrating immune cells.

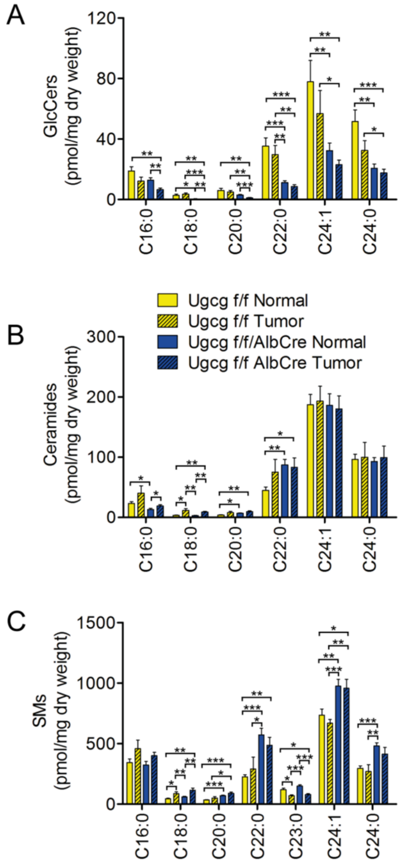

Supplementary Figure 4: Detailed sphingolipid analysis by mass spectrometry. The most relevant sphingolipid species of hepatocytes were measured according to their ceramide proportion, respectively. **(A)**, glucosylceramides were slightly reduced in tumor tissues as compared to normal liver of control- and *Ugcg*-deficient mice. GlcCers in normal liver of *Ugcg*$^{f/fAlbCre}$ mice decreased significantly as compared to controls. **(B)**, ceramides of normal tissue both from control and GSL-depleted livers were not consistently affected by GSL depletion. However, C16 to C20 ceramides increased slightly in tumor tissue of *Ugcg*-deficient- and control mice. **(C)**, particularly the sphingomyelin species with C22 to C24 ceramide proportions increased in *Ugcg*$^{f/fAlbCre}$ mice upon GSL deletion; n=8 per group. Significances, *, $p < 0.05$; **, $p < 0.01$; ***, $p < 0.001$ using student's two-tailed t-test.

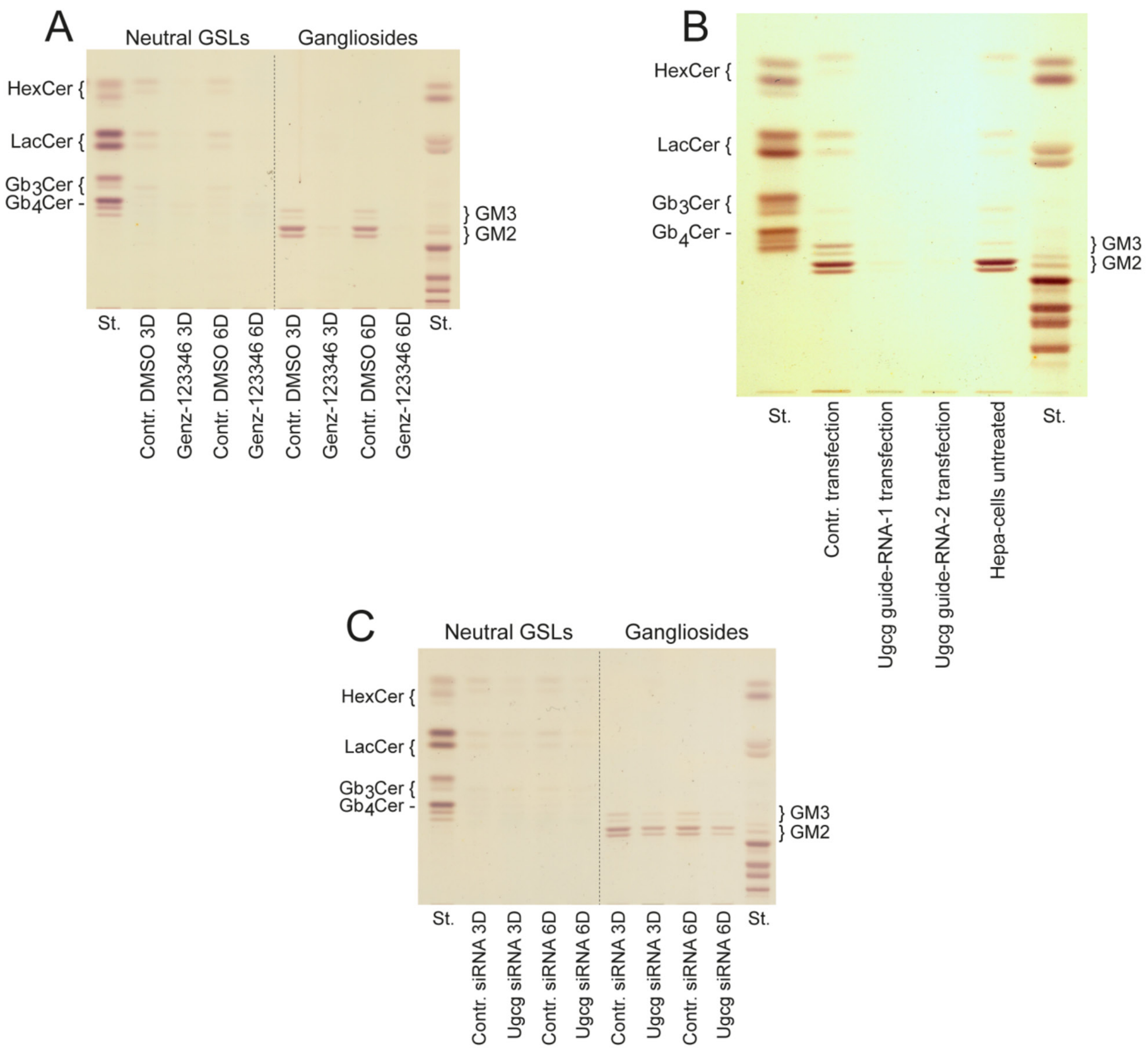

Supplementary Figure 5: Glycosphingolipids are significantly down regulated in Hepa 1-6 cells by chemical or genetic inhibition of Ugcg expression. (A and B), a nearly complete depletion of GSLs could be achieved by treatment of Hepa cells with 5µM Genz for 6 days (A) or transfection with *Ugcg* guide RNA (B). **(C)**, treatment of Hepa cells with specific *Ugcg* siRNA resulted in a GSL reduction of ~50% within 6 days.

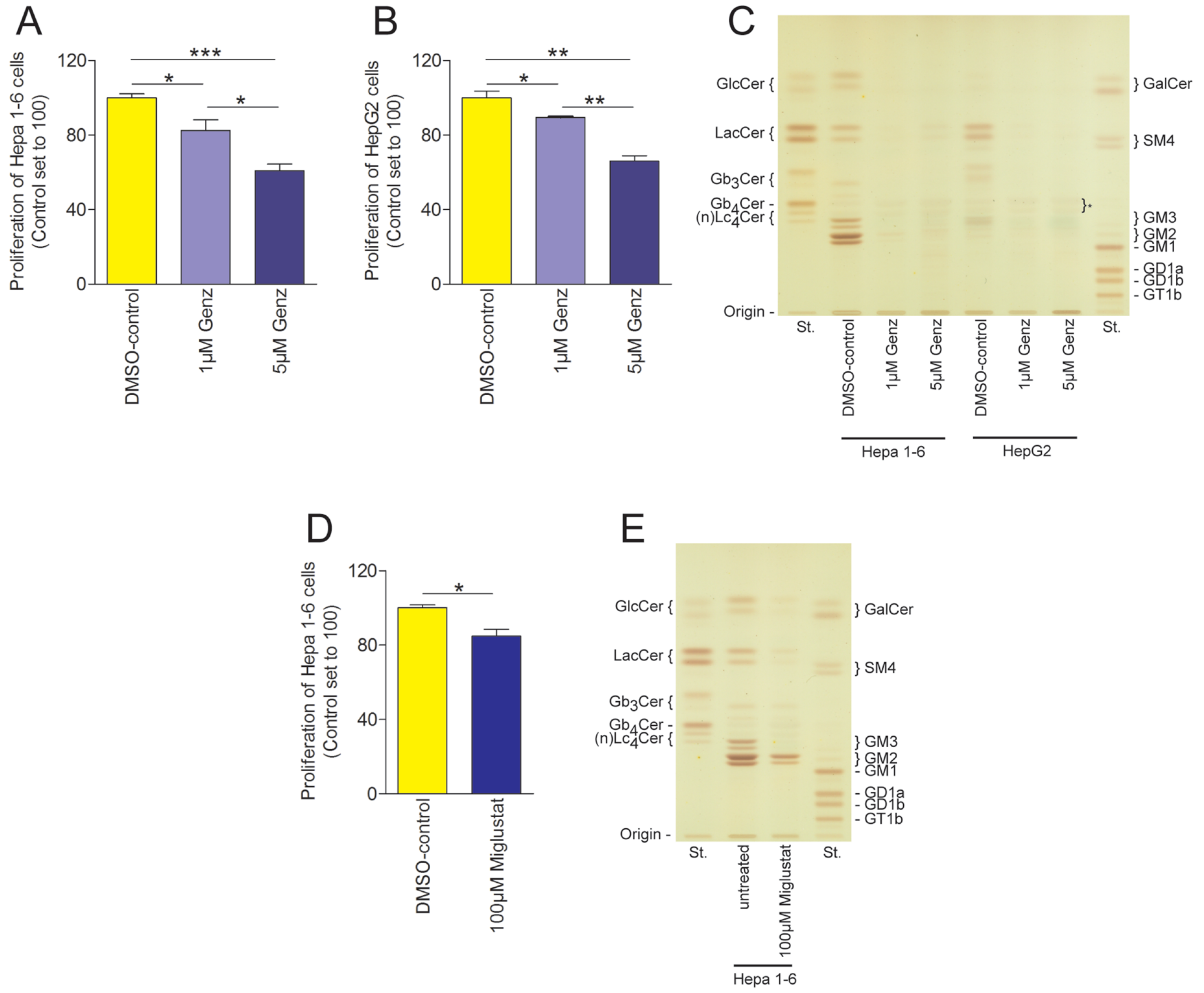

Supplementary Figure 6: The proliferation of HepG2 cells is inhibited by Ugcg-silencing with Genz; Miglustat significantly affects the proliferation of Hepa 1-6 cells. **(A)**, the proliferation of Hepa 1-6 cells significantly decreased by addition of 1µM Genz and even stronger with 5µM Genz to the culture medium. **(B)**, Genz treatment of human HepG2 hepatocellular carcinoma cells resulted in similar effects. **(C)**, TLC of the cell extracts. GSLs are largely depleted by Genz-treatment in both Hepa 1-6- and HepG2 cells. **(D)**, the proliferation of Hepa 1-6 cells significantly decreased in Miglustat-treated cells. **(E)**, TLC of Miglustat-treated Hepa 1-6 cells. GSLs were significantly reduced (>50%) by addition of 100µM Miglustat to the culture medium; n=3 per group. $\}^{*}$, no GSL-positive staining. Significances, *, $p < 0.05$; **, $p < 0.01$; ***, $p < 0.001$ using student's two-tailed t-test.

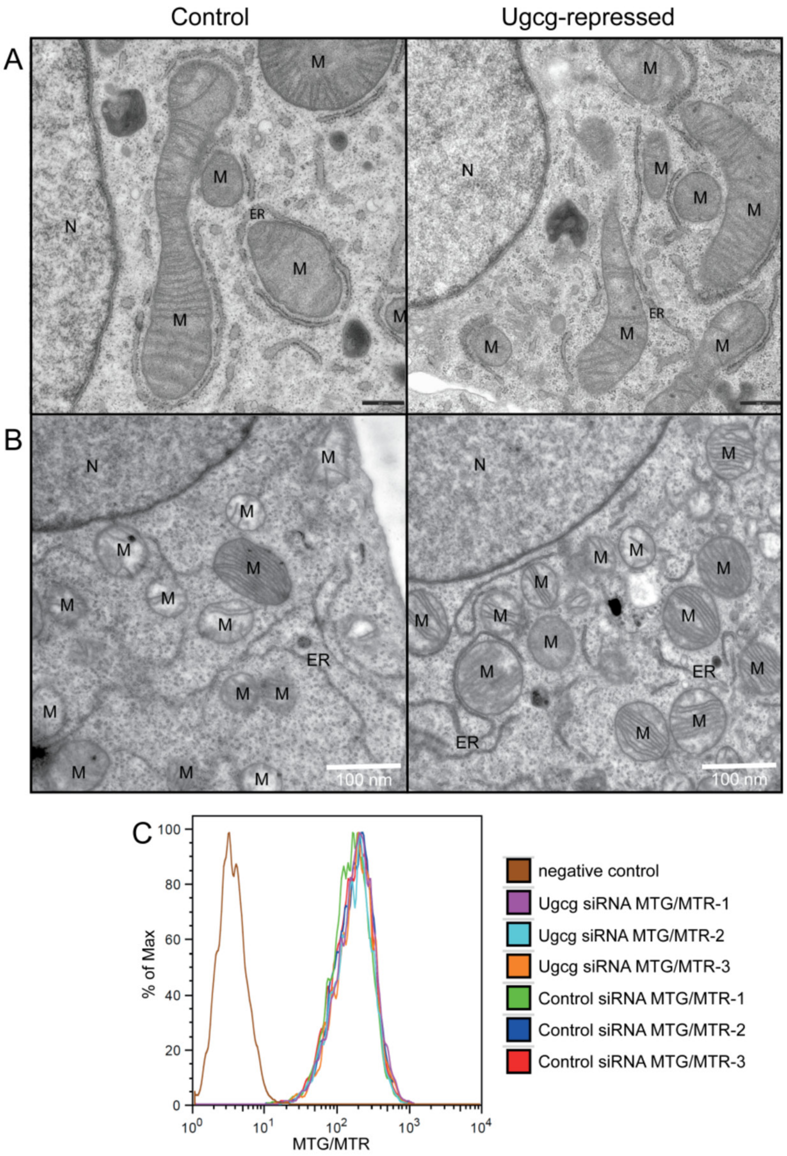

Supplementary Figure 7: Organelles of *Ugcg* siRNA-treated hepatoma cells have an unaltered morphology in comparison to untreated cells. (A and B), cell organelles of Hepa 1-6 cells after *Ugcg* repression with 5µM Genz (A) or Ugcg siRNA (B) displayed a regular ultra-structure. Increased autophagy or apoptosis have not been observed; N, nucleus; M, mitochondria; ER, endoplasmic reticulum. **(C)**, mitotracker green/-red (MTG/MTR) staining indicated regular mitochondrial function in *Ugcg* siRNA-treated as compared to control siRNA-treated hepatoma cells.

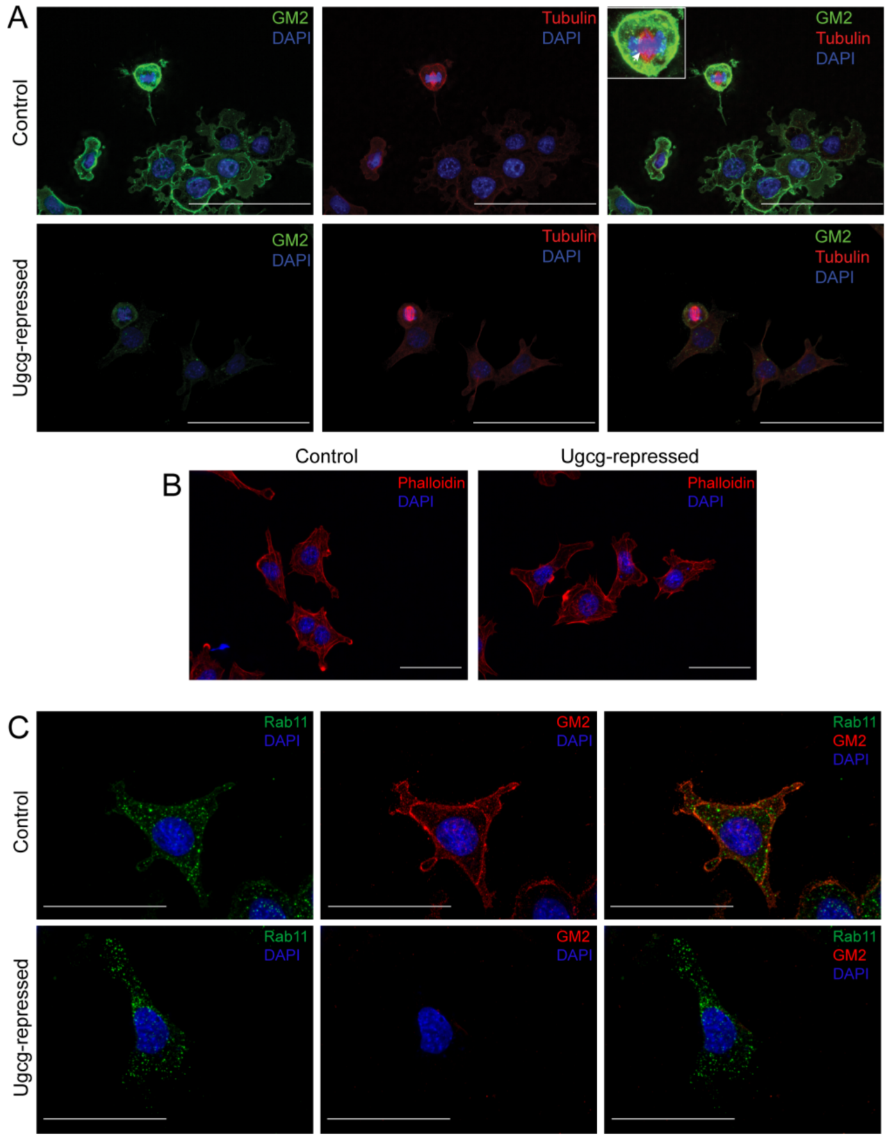

Supplementary Figure 8: GSL depletion does not affect spindle microtubules and the cytoskeleton of Hepa 1-6 hepatoma cells and GSLs do not co-localize with recycling endosomes. **(A)**, GM2 was prominently expressed at the cell membrane during mitosis but did not co localize with tubulin in microtubules (inset, arrowhead). Genz treatment of Hepa cells for 5 days had no impact on microtubule formation (A, lower panel). **(B)**, Hepa cells stained with phalloidin showed a regular occurrence of cellular actin bundles independent from GSL expression. **(C)**, recycling endosomes, labeled by Rab11 immunofluorescence, showed cytosolic distribution in control- (upper panel) and Ugcg-repressed cells (lower panel). GM2 located predominantly at the plasma membrane and consequently did not overlap with Rab11 in the cytosol; scale bars, 50µm.

Supplementary Table1: Antibody dilutions for Western Blot analysis

1st antibody	Host	Dilution	Blocking/ Inkub.	Supplier	Size (kD)
Cyclin D1	m	1:500	5% Milk/TBST	SC-450	34
Cyclin A	rb	1:1000	5% Milk/TBST	SC-596	55
p27	m	1:200	5% Milk/TBST	Cell Signaling 3698	27
p21	rb	1:100	5% Milk/TBST	SC-397	21
ERK 1/2	m	1:500	5% Milk/TBST	BD Biosci. 610123	42/44
Phospho ERK 1/2	m	1:1000	1% BSA/TBS	BD Biosci. 612358	42/44
EGFR	rb	1:1000	5% BSA/TBST	Cell Signaling 4267	170
Phospho-EGFR	rb	1:1000	5% BSA/TBST	Cell Signaling 3777	170
β-Catenin	m	1:2000	5% Milk/TBST	BD Biosci. 610153	92
mTOR	rb	1:1000	5% BSA/TBST	Cell Signaling 2972	289
Phospho-mTOR	rb	1:1000	5% BSA/TBST	Cell Signaling 2974	289
Akt	rb	1:2000	5% BSA/TBST	Cell Signaling 9272	60
Phospho-Akt	rb	1:500	5% BSA/TBST	Cell Signaling 4058	60
Actin	rb	1:500	5% Milk/TBST	SC-1616-R	42

m, mouse; rb, rabbit; SC, Santa Cruz Biotech.; BD, Becton Dickinson Biosc.
